# Application of cold argon plasma on germination, root length, and decontamination of soybean cultivars

**DOI:** 10.1186/s12870-024-04730-4

**Published:** 2024-01-22

**Authors:** Khadijeh Sayahi, Amir Hossein Sari, Aidin Hamidi, Bahareh Nowruzi, Farshid Hassani

**Affiliations:** 1 Department of Physics, Faculty of Converging Sciences and Technologies, Science and Research Branch, Islamic Azad University, Tehran, Iran; 2 Seed and Plant Certification and Registration Research Institute (SPCRI), Agricultural Research, Education and Extension Organization (AREEO), Karaj, Iran; 3 Department of Biotechnology, Faculty of Converging Sciences and Technologies, Science and Research Branch, Islamic Azad University, Tehran, Iran

**Keywords:** Cold plasma, Argon, Soybean, *Aspergillus flavus*, *Fusarium solani*, Seed germination, Root length, Electrical conductivity

## Abstract

Applying cold discharge plasma can potentially alter plants' germination characteristics by triggering their physiological activities. As a main crop in many countries, soybean was examined in the present study using cultivars such as Arian, Katoul, Saba, Sari, and Williams in a cold argon plasma. This study has been motivated by the importance of plant production worldwide, considering climate change and the increasing needs of human populations for food. This study was performed to inspect the effect of cold plasma treatment on seed germination and the impact of argon plasma on microbial decontamination was investigated on soybeans. Also, the employed cultivars have not been studied until now the radicals generated from argon were detected by optical emission spectrometry (OES), and a collisional radiative model was used to describe electron density. The germination properties, including final germination percentage (FGP), mean germination time (MGT), root length, and electrical conductivity of biomolecules released from the seeds, were investigated after the plasma treatments for 30, 60, 180, 300, and 420 s. The decontamination effect of the plasma on *Aspergillus flavus* (A.flavus) and *Fusarium solani* (F.solani) was also examined. The plasma for 60 s induced a maximum FGP change of 23.12 ± 0.34% and a lowest MGT value of 1.40 ± 0.007 days. Moreover, the ultimate root length was 56.12 ± 2.89%, in the seeds treated for 60 s. The plasma exposure, however, failed to yield a significant enhancement in electrical conductivity, even when the discharge duration was extended to 180 s or longer. Therefore, the plasma duration of 180 s was selected for the blotter technique. Both fungi showed successful sterilization; their infectivity inhibition was 67 ± 4 and 65 ± 3.1%, respectively. In general, the cold plasma used for soybeans in the present study preserved their healthy qualities and reduced the degree of fungal contamination.

**Supplementary Information:**

The online version contains supplementary material available at 10.1186/s12870-024-04730-4.

## Introduction

Cold plasma comprises tiny particles like neutral molecules, atoms and electrons [1]. Despite the high temperature of these particles, the temperature of the substrate treated by cold plasma is close to the ambient temperature, ranging from 25 °C–100 °C [2] or as low as 35 °C [3]. This method is relatively fast and eco-friendly to induce seed germination, growth, and sterilization [4]. In this way, the plasma type attracts significant interest in the food industry. The biotic factors such as drought, temperature, nutrient deficiency, and osmotic ones lead to poor crop growth. Different physical and chemical methods have been employed to induce germination, including magnetic treatment, genetic manipulations, sunlight, ultraviolet light, hot water soaking, hormonal priming and chemical methods [5]. However, apart from being time-consuming, they are not economical, need exhausting work, and are always companying with environmental pollution. In contrast, this method is quick, cost-effective, eco-friendly, and potent enough to induce seed germination besides decontaminating [4]. Historically, this method in 1960 was introduced as a sterilizing method and in this manner, a patent was registered in 1968 [6]. If the penetration depth of UV photons is compared with nonthermal plasma, their powers are 1 and 10 µm, respectively [7]. As a function of the high penetrating potential of this method, cold plasma can destroy fungi and sporulated bacteria, and its mechanism is related to damage to DNA in the chromosomes. This strategy accompanies the oxidation of biomolecules inside plants, such as lipids and proteins, making unsaturated fatty acid peroxides and oxidized amino acids. This process creates morphological changes and, at last, microorganism lysis. These observations explain this method's efficiency despite repair mechanisms in microbial cells in other sterilization methods such as UV [8]. A group reported that using oxygen and nitrogen cold plasma on carrots for 4–5 min decreased spoilage microflora significantly, and thus, quality retention was enhanced [9]. Another research confirmed that by cold plasma for 5–20 min, the contamination of legumes by Aspergillus spp. and Penicillum spp. decreased significantly, and their germination potency was intact [10]. A similar investigation approved that the microbial pollution of brown rice to aerobic bacteria was reduced considerably after cold plasma exposures [11].

Numerous examinations have confirmed the positive effect of cold plasma on plant growth performance. The mechanism of this approach is to activate physiological functions in plasma-treated seeds. One study showed that cold plasma increased the expression of essential enzymes such as peroxidase, polyphenol oxidase, and phenylalanine ammonia-lyase from tomato seeds [12]. In another group, the sunflower seeds treated for 7 min before sowing increased germination, leaf weight, and seedling growth. In addition, gibberellin and abscisic acid production was modified to increase germination [13]. After cold plasma treatment, two buckwheat cultivars indicated higher photosynthesis and metabolite/mineral production [14]. One of the mechanisms of plasma to increase plant growth is related to its changes to seed-wetting properties [15]. Another method involves eroding the surface of the seeds with free radicals [16]. An investigation revealed that in plasma–irradiated rice seeds (*Oryza sativa L.*), hypermethylation of *OsNCED5* promoter and hypomethylation of *OsAmy1C* and *OsAmy3E* promoters occurred to restore them under environmental stress [17]. Additionally, the dormancy phase of radish seeds (*Raphanus sativus*) was influenced by plasma, and their germination was accelerated during the early stages of harvest. The formation of abscisic acid and gibberellin was changed in the plasma–treated groups, and in contrast to the higher production of the former, the latter decreased [18]. The Plasma processing time, voltage, and power must be optimized according to the seed type and environmental conditions [18]. Therefore, varying seed sources may behave differently after plasma discharge. The pressure of the gas and the environment’s temperature alter the result of this technology by different energy and density of the radicals. However, cold plasma is carried out at atmospheric pressure, and thus, the interaction of the radical species and seeds can take place outdoors without pressure restrictions [19]. Another plasma factor to consider is related to carrier gas type. In one assessment, modified atmosphere gas (MA65) (65% O_2_, 30% CO_2_, 5% N_2_) and air were selected to evaluate their effect on aflatoxin degradation. The observations have confirmed that the air in this application depends more on the ambient humidity (%).

In contrast, MA65 operates independently of environmental parameters such as humidity [20]. However, the nature of the working gas may not make a significant difference in some applications. Gases including N_2_, CO_2_, Ar, O_2_, and air did not differ considerably in mycotoxin degradation [21]. Compared with nitrogen, argon has a higher inactivation value for some enzymes, such as lipase and lipoxygenase [22]. Therefore, the outcomes of plasma-seed interactions can be complex and must be evaluated against many parameters. Noble gases such as argon are expensive [23], with high thermal conductivity, emitting UV light to kill fungi and bacteria [24]. One research group reported that argon plasma limits the growth of S. aureus for 5 min [25]. Seeds are treated with fungicides and insecticides to increase their survival. These chemical substances are now considered the major factors that cause allergies after eating plants [26]. Thus, the preservation of the nourishing properties of seeds is a significant concern. It has been shown that the free radicals generated by plasma treatment limit the use of this method.

On the other hand, cold plasma can limit their contamination without exposing these compounds to toxic effects. Therefore, as a decontamination strategy, this method can also increase seed germination. Regarding this, the employment of cold plasma to diminish microorganism infection may help the germination and following growth phases of plants. In an identical mechanism, the coat of seeds is broken after cold plasma treatments, and oxygen or moisture quickly transfers into the embryo, facilitating seed germination. Signal molecules such as growth factors and enzymes are activated and break down seed dormancy. Therefore, the treated seeds are germinated sooner than the non-treated ones. Among various crops, soybean is a main one in the food industry in many countries [27, 28], and fresh quality is needed for the feed industry [29]. Additionally, its yield is affected when environmental conditions are not optimized [30].

In this work, 4 soybean cultivars belonging to 2 classes were selected to evaluate the efficiency of argon plasma. Different seed exposure times were applied, and final germination percentage (FGP), mean germination time (MGT), and root length were measured. Other properties examined in the present study were the electrical conductivity of the seed solutions and the efficiency of plasma decontamination.

## Methods and materials

### Cold plasma apparatus description

**Fig. 1:**
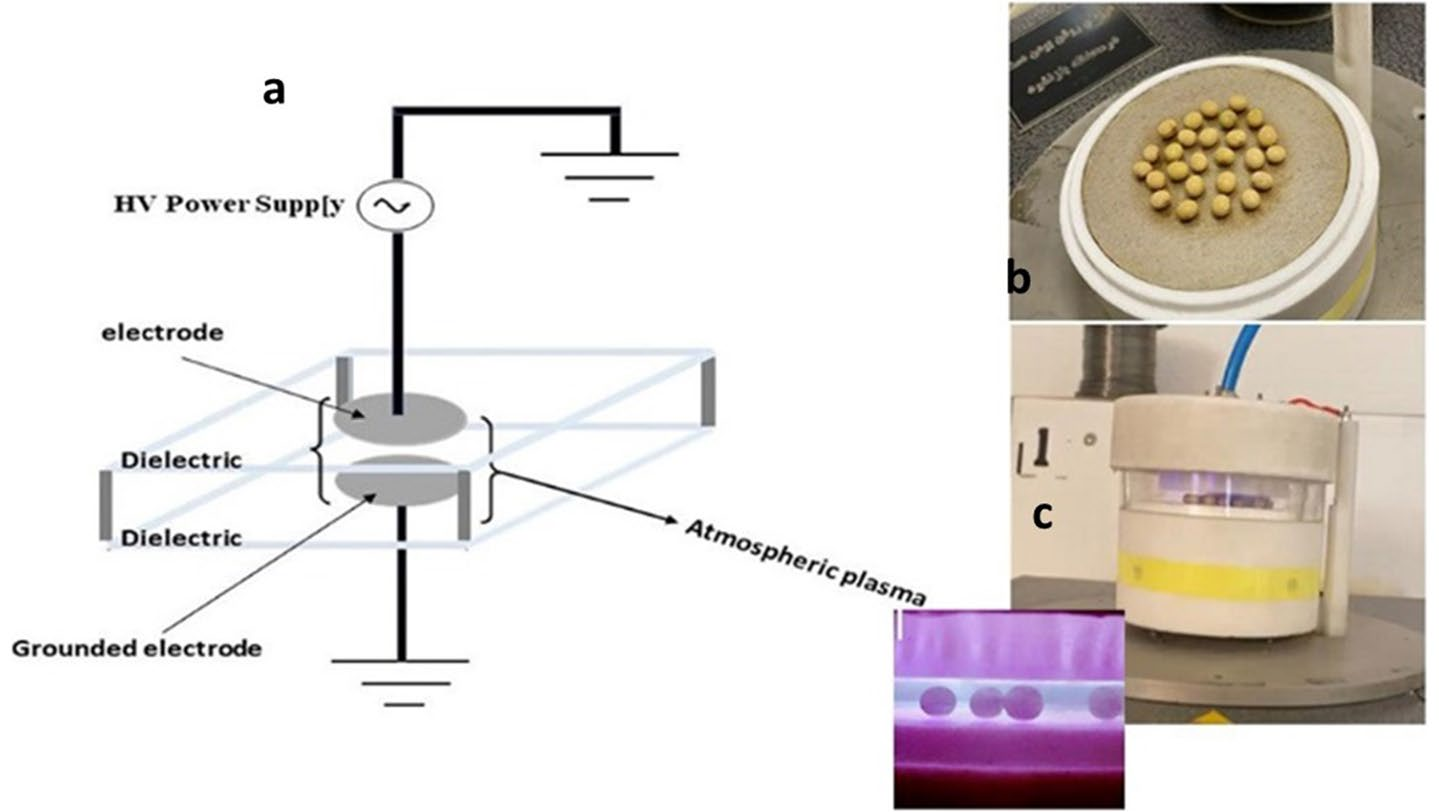
**a** A schematic view of cold plasma apparatus, **b** The position of seeds on the apparatus, and **c** The apparatus when the plasma was turned on and applied to seeds. The enlarged part indicates the filamentary cold plasma inside the system

The dielectric barrier discharge (DBD) plasma (High Tech Company, 0045, Tehran, Iran) contained two flat aluminum electrodes with dimensions of 45, 6.5, and 0.02 cm as the length, width, and thickness. A mica-derived insulating sheet is positioned between the electrodes to limit the current flow to µA. The dimensions of this sheet were 60, 12, and 0.01 cm as the length, width, and thickness, respectively. Also, a plexiglass shield with a width of 0.03 cm was fixed between the electrodes. The gas supply used to develop plasma was argon. Due to the atmospheric pressure in the plasma, a vacuum pump was not needed. The energy dissipation occurs due to collisions of charged particles [31]. The corresponding value in the present study was measured in electrodes using a thermocouple, and the temperature was almost 40° C. A schematic view of the apparatus is shown in Fig. 1. The cold plasma system was closed using an acrylic chamber containing a gas inlet and outlet. During plasma treatments, the distance between the tip of the plasma plume and the seeds was fixed at 20 mm. The current supply was connected to an alternating current with a primary voltage of 220 V. The voltage and frequency applied on the plasma plume were 5 kV and 8 kHz with a power of 100 W. The feed gas was argon, and the flow rate of this gas was 2 L/min. In accordance with the properties of this apparatus, the voltage was 5 kV with a frequency of 8 kHz. Also, the maximum current was 10 mA. Therefore, the power consumption of this system by using Watt's law was calculated as below:

$$\mathrm{P }=\mathrm{ V }\times \mathrm{ I}$$

where p, V and I are power, voltage and current, respectively. Therefore, the power value is resulted as 100 W.

Regarding references, the power consumption of an experimental cold plasma on lab scales is from 15 to 900 W [23]. This scale of cold plasma is usually employed to modify surface materials such as seeds through a surface etching mechanism [32].

### Examination of developed radicals by DBD

**Fig. 2:**
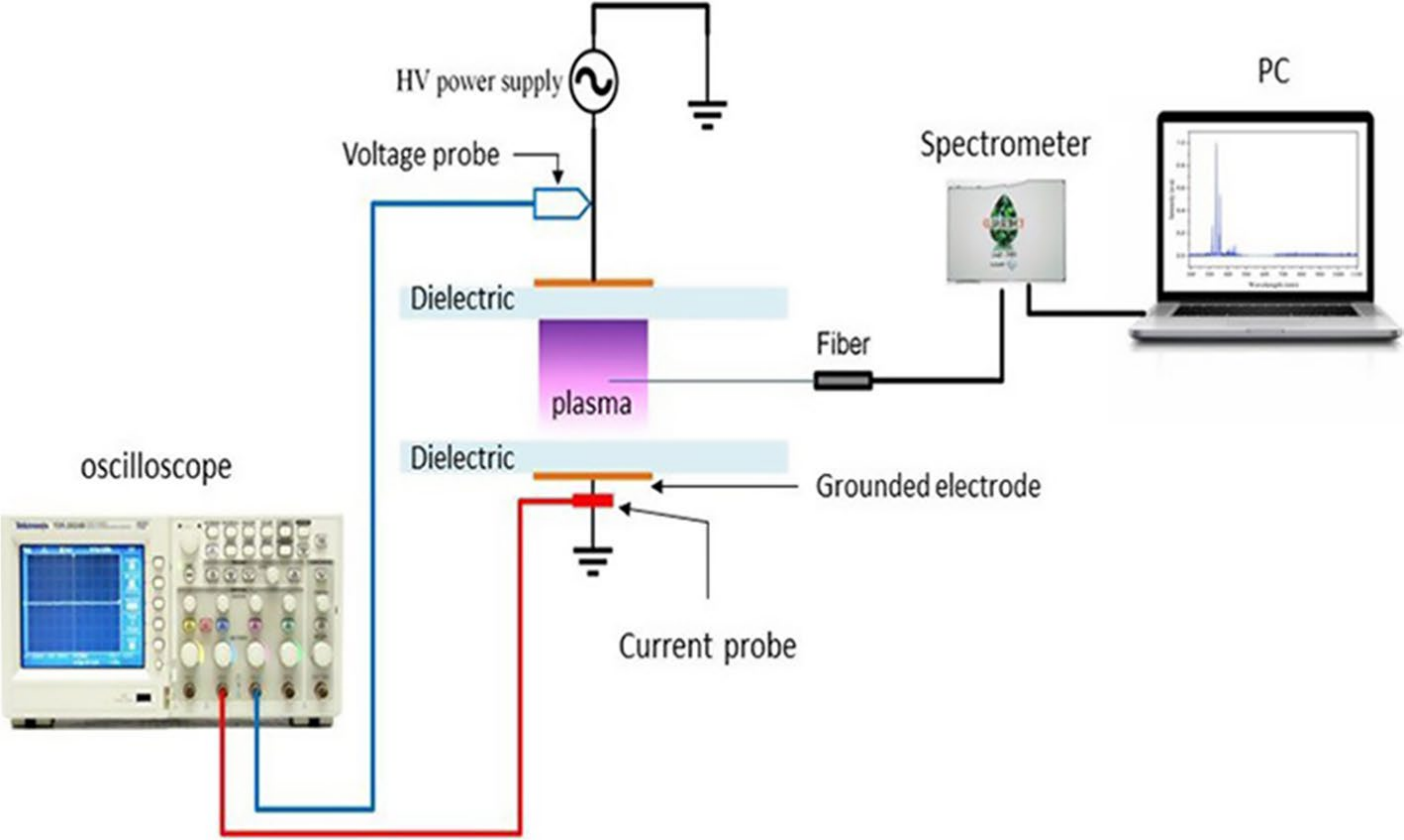
Schematic of the experimental setup for optical emission spectroscopy

**Fig. 3:**
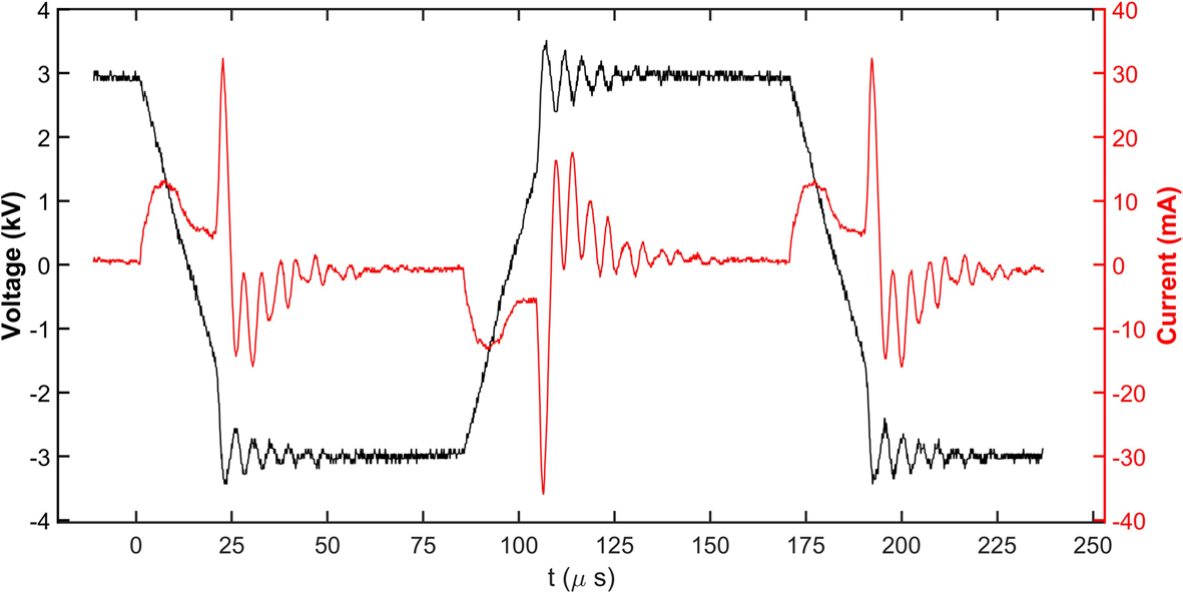
Electrical signal (voltage and current) of the argon DBD

Optical emission spectroscopy (OES, Ocean Optics, HR4000CG-UV-NIR, USA) was used to detect the UV–visible emission spectrum associated with the generated free radicals (Fig. 2). Voltage values were measured directly with a high voltage probe (P6015A Tektronix HV) and current using a digital oscilloscope (Tektronix MSO4032) equipped with a TCP202 Tektronix current probe. Figure 3 shows the voltage-current waveforms for the DBD plasma with a fixed Ar flow value of 2 L/min and at a peak applied voltage of 5 kV.

### Argon collisional-radiative model (CRM) by DBD

Using CRM, the behavior of the plasma was determined by predicting electron temperature and density. Besides electronic excitation from its ground state, this model also considers excitation transition, radical recombination, and ionization [33]. The intensity of emission line ${(I}_{ji}$) excited to the upper state (from *i* to *j* state), is calculated according to the equation below [34]:

$${I}_{ji}={E}_{ji}{N}_{j}{A}_{ji}\left(W/{cm}^{3}\right)$$

where ${E}_{ji}$ is the energy difference between ${E}_{j}$ and ${E}_{i}$ (${E}_{j}> {E}_{i}$), ${A}_{ji}$ is the possibility of transfer and ${N}_{j}$ is the population density of the upper state (j).

McWhirter’s criterion describes when a plasma state is classified as a local thermodynamic equilibrium (LTE). According to LTE, the resultant spectral intensity is equivalent to the electron density [35]. Considering this, the slope of the Boltzmann plot used to calculate the approximate temperature depends on the density of the excited electrons. The dependency between electron density and temperature is explained by the following formula [36]:

$${N}_{e}\ge 1.6\times {10}^{12}{T}^\frac{1}{2}{\left(\Delta E\right)}^{3}$$

where ${N}_{e}$ (cm^−3^) is the electron density, T is the plasma approximate temperature, and $\Delta E$ is the energy difference between the two states.

The difference of the energy states is used with the following formula for each state to determine the approximate temperature [37, 38]:

$${I}_{\mu }=\frac{{{h}_{c}A}_{ji}{{\text{g}}}_{{\text{j}}}}{{\lambda }_{\mu }{Q}_{s}\left(T\right)}{\text{exp}}\frac{-{E}_{j}}{{K}_{B }{T}_{e}}$$

Herein, ${I}_{\mu }$ is the integral intensity of the emission line ( *j*) and${\lambda }_{\mu }$, h_c_, A_ji_, g_j_, E_j_, Q_s_, T, K_B,_ and *T*_*e*_ are wavenumber, plank constant, probability of spontaneous emission transmission from the upper-level *j* to the lower level *i* (Einstein’s coefficient), the statistical weight of the emitted upper-level *j*, excitation energy, appropriate partition function, approximate plasma temperature, Boltzmann constant and electron temperature.

Considering the ratio of the two states, the line intensity ratio (*L*) formula resulted in measuring approximate electron temperature:

$$L=\frac{{I}_{1}}{{I}_{2}}=\frac{{{\text{g}}}_{1}}{{{\text{g}}}_{2}}\frac{{A}_{1}}{{A}_{2}}\frac{{\lambda }_{2}}{{\lambda }_{1}} {e}^{\left[-(\frac{{E}_{1}-{E}_{2}}{{K}_{B }{T}_{e}})\right]}$$

The values related to the emission lines, and other spectroscopic parameters were taken from the NIST atomic database [39].

### Seed treatment by DBD

Healthy soybeans comprised five cultivars: Arian, Katoul (Katool), Sari, Saba, and Williams. Each cultivar was evaluated under two classes of registered and certified types. These seeds were purchased from the Seed and Plant Certification and Registration Institute, Karaj, Tehran. Before the evaluations, the seeds were examined to discard some that were not healthy macroscopically. Twenty-five seeds were considered for any treatment condition to reduce unwanted and unpredicted parameters. For assays, the seeds were randomly selected for the groups. The control group (without any plasma treatment) was considered representative of initial fungal contamination, and all treatment groups were compared to the control one. To keep the seed contamination level, all seed groups, were kept at 4℃ to inhibit fungal growth. This low temperature can guarantee a fixed level of remaining fungal contamination that was not destroyed by cold plasma. On the other hand, the contamination of the control group was not allowed to grow. The seeds were transferred to a growth temperature of 25 ± 1.5℃ to perform microbial analysis via blotter method. The only soybean cultivars that can be cultivated in Iran are Arian, Katoul (Katool), Sari, Saba, and Williams. Katoul cultivars are the most essential cultivars of soybean cultivated in Golestan province, Sari, and the most critical cultivar of soybean grown in Mazandaran province, while the most important cultivars of soybean developed in Ardabil provinces (Moghan) are Williams, Saba, and Arian. Golestan and Ardabil provinces are ranked first and second, respectively, in terms of cultivation area, while regarding grain production, Ardabil is the first, and Golestan is the second. The dimensions of the seeds were collected in Table 1. The seeds were in direct contact with the plasma developed using argon gas with a voltage and frequency of 5 kV and 8 kHz, respectively. The current was almost 1 µA, and the volumetric flow rate of argon gas was maintained at 2000 ml/min. The space between the seeds was 2 mm from each side In accordance with Fig. 1, the position of the seeds is clear. Exposure times and temperature were 30, 60, 180, 300, and 420 s at 25° C. Moreover, non-treated seeds were considered as the control group in the nest-factorial design model with 4 replicates. The effect of the DBD treatments on the respective seeds was examined after 8 days.

**Table 1:** The dimensions and weights of the seeds were used in the present study

Cultivar	Class (within cultivar)	The size of each seed (mm)	Weight of each seed (g)
**Sari**	Registered	4.55	0.196
	Certified	4.25	0.175
**Saba**	Registered	5.25	0.165
	Certified	5.05	0.146
**Arian**	Registered	5.35	0.249
	Certified	5.15	0.219
**Katoul**	Registered	4.95	0.198
	Certified	4.80	0.154
**Williams**	Registered	4.75	0.161
	Certified	4.60	0.132

### Seed harvesting after plasma treatment by DBD

Seeds, both treated and non-treated, were germinated on filter papers with 30 × 45 cm^2^ dimensions. The total number of seeds was 200, divided into 25 groups, and each group repeated 8 times to design the experiment in a random model. The seeds were layered on paper and moistened with 10 ml of distilled water. On the following days, 5 ml of water was added to provide the necessary humidity of 85% for their germination. Samples were harvested at 25 ± 1.5℃ in a germinator (Seedburo Equipment Co., Chicago, IL) under fluorescent lighting for a 12 h light and 12 h dark cycle. During the entire harvest period of 8 days, the distance between the light source and the seeds was fixed at 40 cm.

### Examination of seed germination criteria after plasma treatment by DBD

Healthy seed quality, including final germination percentage (FGP, %), mean germination time (MGT, day), fungal infection, and root length (cm), was recorded after 8 days. The value of FGP and MGT was calculated according to the formula below [5, 40, 41]:

$$\mathrm{FGP}\;(\%)\;=\;\lbrack\mathrm{Number}\;\mathrm{of}\;\mathrm{seeds}\;\mathrm{geminated}\;\mathrm{after}\;\mathrm n\;\mathrm{days}\;/\;\mathrm{Total}\;\mathrm{number}\;\mathrm{of}\;\mathrm{seeds}\rbrack\;\times\;100$$

$$\mathrm{MGT}\;(\mathrm{day})\;=\;\lbrack\mathrm{Number}\;\mathrm{of}\;\mathrm{seeds}\;\mathrm{geminated}\;\mathrm{after}\;\mathrm n\;\mathrm{days}\;\times\;\mathrm{Total}\;\mathrm{number}\;\mathrm{of}\;\mathrm{days}\rbrack\;/\;\mathrm{Total}\;\mathrm{number}\;\mathrm{of}\;\mathrm{seeds}\;$$

Moreover, variations in FGP could be another helpful factor in distinguishing treatment groups by subtracting their FGPs. The groups with negative and positive values were considered inefficient and efficient. In addition to reporting root length (cm), to improve their comparison, improvement rate (%) was used to describe the positive difference between the treated and non-treated groups according to the formula down here:

$$\mathrm{Improvement}\;\mathrm{rate}\;(\%)\;=\;\lbrack\mathrm{Root}\;\mathrm{length}\;\mathrm{after}\;\mathrm n\;\mathrm{days}\;\mathrm{of}\;\mathrm{treated}\;\mathrm{seed}\;-\;\mathrm{Root}\;\mathrm{length}\;\mathrm{after}\;\mathrm n\;\mathrm{days}\;\mathrm{of}\;\mathrm{non}-\mathrm{treated}\;\mathrm{seed}\rbrack\;/\;\mathrm{Root}\;\mathrm{length}\;\mathrm{after}\;\mathrm n\;\mathrm{days}\;\mathrm{of}\;\mathrm{non}-\mathrm{treated}\;\mathrm{seed}\;\times\;100$$

The experimental design performed in this study is a factorial nested model. For this method, a mixed model analysis of covariance was calculated for each property, including FGP, MGT, and root length. These calculations distinguish the effects of cultivar, class, and plasma time in addition to the combinations of cultivar and class with time on the corresponding physiological activities of the seeds.

### Electrical conductivity of solutions containing treated seeds by DBD

For this assay, 250 ml of deionized water was stored for 24 h at 20℃. The seeds were weighed and soaked in the water. Then, after 24 h, their electrical conductivity was recorded with a conductivity meter (Mettler Toledo FE38-Meter, Zurich, Switzerland), and the values were reported as follows [42]: 

$$\text{Electrical conductivity}\ (\upmu{\text{s}}.\text{cm}^{-1}.\text{g}^{-1}) = \text{Electrical conductivity of a sample}\ (\upmu{\text{s}}.\text{cm}^{-1})\; /\; \text{Seed weight}\ (\text{g})$$

### Microbial analysis of seeds treated by DBD

The blotter method examined the effect of plasma on the inactivation of fungal growth [43]. Two fungi were employed: Aspergillus flavus (A.flavus) and Fusarium solani (F.solani). For this assessment, after the plasma treatment, the fungi were harvested on all seed groups, including treated and non-treated. After wetting, the filter papers containing the treated and non-treated seeds were placed clockwise at 25° C for 7 days in a petri dish. As with the standard harvesting procedure described above, the light and dark conditions were shifted every 12 h, and the light source was an ultraviolet lamp at a distance of 40 cm. The infected seeds were determined using a stereo microscope (Olympus, Japan). Moreover, contamination removal (%) is a valuable criterion to compare the resistance of seeds to microorganisms. Therefore, the value was computed using the formula below:

$$\mathrm{Contamination}\;\mathrm{removal}\;(\%)\;=\;\lbrack\mathrm{Sum}\;\mathrm{contamination}\;\mathrm{of}\;\mathrm{treated}\;\mathrm{group}\;-\;\mathrm{Sum}\;\mathrm{contamination}\;\mathrm{of}\;\mathrm{non}-\mathrm{treated}\;\mathrm{group}\rbrack\;/\;\mathrm{Sum}\;\mathrm{contamination}\;\mathrm{of}\;\mathrm{non}-\mathrm{treated}\;\mathrm{group}$$

### Statistical section

For statistical analysis, sigma plot software was employed, and Student’s t-test compared the differences between the two groups. ANOVA was used to examine for variance in a nested factorial design involving mixed factors. For the t-test and ANOVA, the p-values less than 0.05 and 0.01 were considered significant, respectively, and all data were reported as mean ± standard deviation (SD).

## Results

### OES assessment to detect radical species

**Fig. 4:**
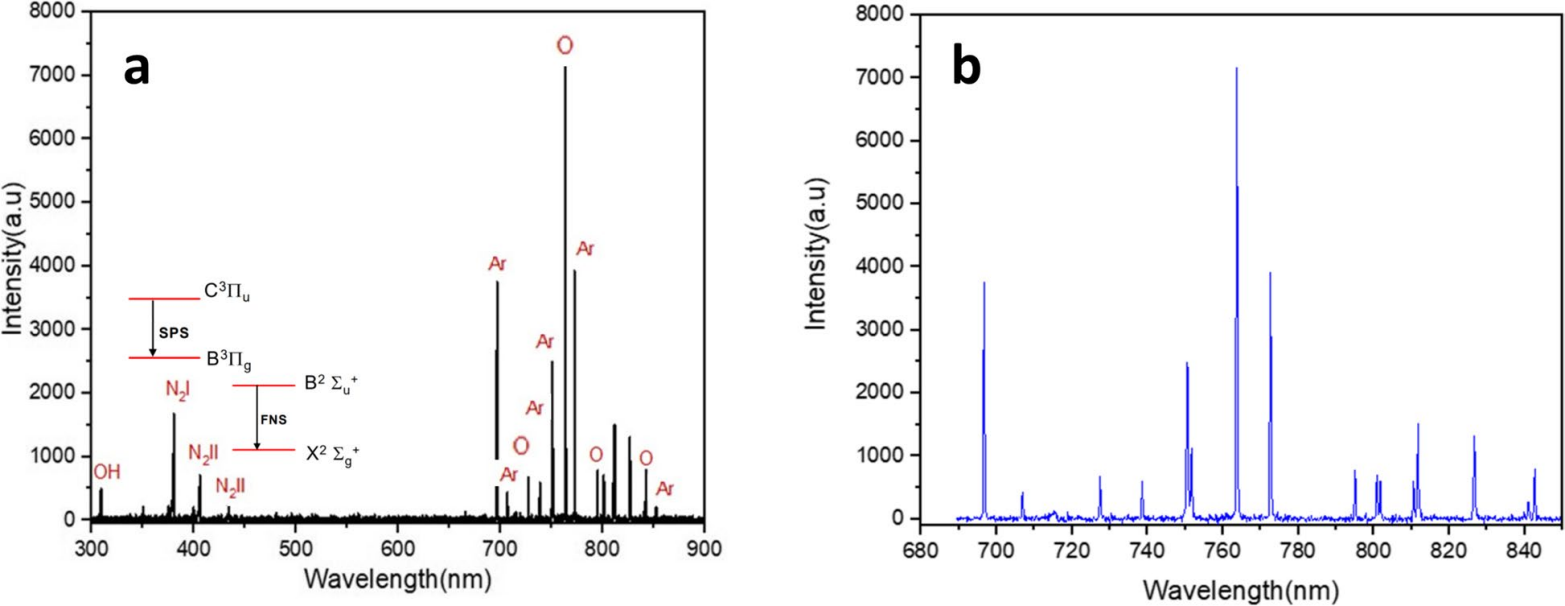
**a** Emission spectrum of argon plasma with voltage and frequency of 5 kV and 8 kHz at atmospheric pressure and **b** Theoretical (CRM) spectral line intensities of argon plasma

The DBD's efficiency was examined here by evaluating its free radicals via the OES method in the UV–visible wavelength range of 300–900 nm with argon gas. This assessment was done in the presence of seeds to ensure the development of radical species at unique plasma parameters, including frequency, voltage, power and time to treat the seeds. The results have been reported in Fig. 4a. The filamentary mode of cold plasma is usually developed by inert gases like argons that are less expensive. Therefore, the grown plasma filaments were distributed throughout the whole volume of the respective tube. Based on the curve, most peaks are exposed between 700–900 nm. The free radicals developed after the plasma treatment were OH·, N_2_ I·, N_2_ II·, O·, and Ar·.The emission wave at 309 nm was representative of the OH radical [44]. The peaks at 375 [45] nm are the representative of second positive system of N_2_ (N_2_ I, 2 + , SPS). Moreover, the first negative system of N_2_ (N_2_ II, 1-, FNS) was detected at 405 [46] and 427 [47] nm. Moreover, argon radicals made other high-intensity peaks at 696, 707, 739, 750, 773, and 852 nm [48]. Additionally, the radicals originated from molecular oxygen's atomic and atmospheric A-band transitions were detected at 844 and 760 nm, respectively [49]. The 760 nm band is associated with the O–O magnetic dipole transitions of O_2_ [50]. Another peak at 725 nm is related to atomic oxygen radicals [51].

### Argon CRM to measure electron temperature

**Fig. 5:**
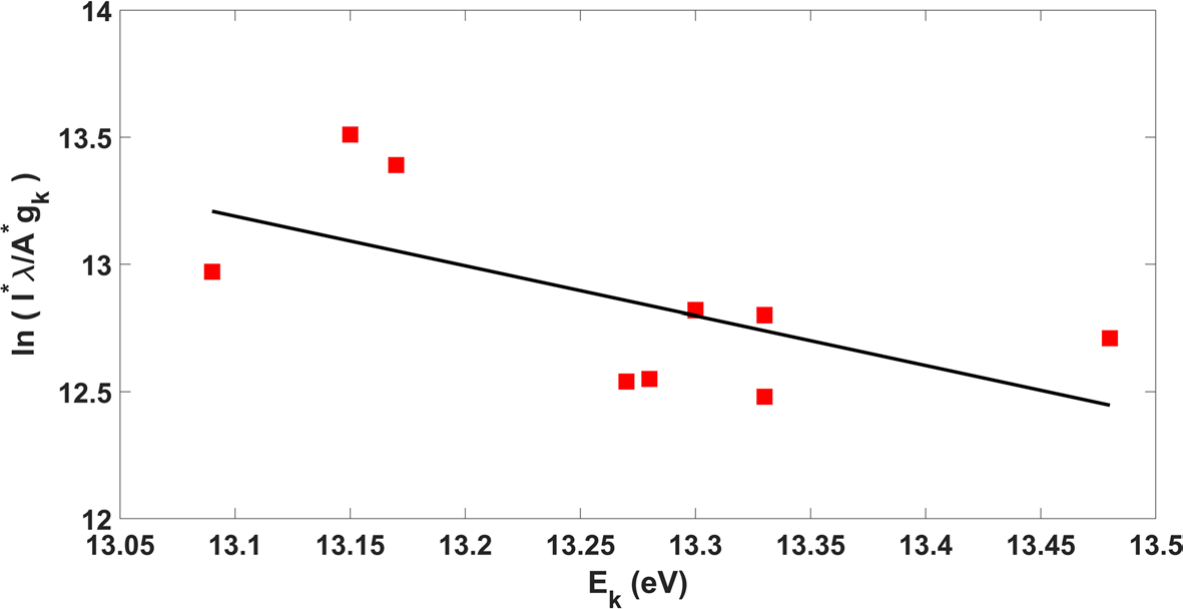
Boltzmann plot from ArI lines produced by plasma DBD

The McWhirter criterion was recruited to calculate the approximate electronic temperature by considering the LTE state for the non-thermal argon plasma in the present study. In this state, the approximate temperature of the excited electrons and also, the electron density describes the properties of the plasma evolved [52] and the intensity of the state energies according to the Atomic Spectroscopy Database at the National Institute of Standards and Technology [53, 54], was used to determine the temperature of the electrons. The relevant observation of CRM argon has been indicated in Fig. 4b. Following Table 2, 696.543 and 794.81 nm states possess energy values of *E*_*j*_ = 13.33 and *E*_*i*_ = 11.55 eV, respectively. These states ($${I}_{1}$$ and $${I}_{2})$$ were selected due to their highest energy difference (NIST atomic database). Finally, by inserting the values of the statistical weight (g), Einstein’s coefficients (A), Boltzmann coefficient (*K*_*B*_), and smallest and largest wavelengths ($$\lambda$$), the approximate temperature was estimated to be *T*_*e*_ = 0.7178 eV which is equivalent to 9097° K. Figure 5 shows the Boltzmann plot.

**Table 2:** Argon energy levels which were considered in CRM model

𝐄𝐦𝐢𝐭𝐭𝐞𝐝 𝐰𝐚𝐯𝐞𝐥𝐞𝐧𝐠𝐭𝐡 (λ 𝐢𝐧 ***nm***)	**Spectral Transition**	**Relative** Intensity (a. u.)	**Ej (eV)**	**Ei (eV)**
696.543	^1^ ${P}_{1}\to$ ^3^ ${P}_{2} {2p}_{2}\to {1s}_{5}$	10,000	13.33	11.55
706.722	^3^ ${P}_{2}\to$ ^3^ ${P}_{2} {2p}_{3}\to {1s}_{5}$	10,000	13.30	11.55
727.294	^1^ ${P}_{1}\to$ ^3^ ${P}_{1} {2p}_{2}\to {1s}_{4}$	2000	13.33	11.62
750.387	^1^ ${S}_{0}\to$ ^1^ ${P}_{1} {2p}_{1}\to {1s}_{2}$	20,000	13.48	11.83
751.465	^3^ ${P}_{0}\to$ ^3^ ${P}_{1} {2p}_{5}\to {1s}_{4}$	15,000	13.27	11.62
772.376	^3^ ${D}_{1}\to$ ^3^ ${P}_{2} {2p}_{7}\to {1s}_{5}$	15,000	13.15	11.55
794.818	^3^ ${P}_{1}\to$ ^3^ ${P}_{0} {2p}_{4}\to {1s}_{3}$	20,000	13.28	11.72
800.616	^3^ ${D}_{2}\to$ ^3^ ${P}_{1} {2p}_{6}\to {1s}_{4}$	20,000	13.17	11.62
801.479	^3^ ${D}_{2}\to$ ^3^ ${P}_{2} {2p}_{8}\to {1s}_{5}$	25,000	13.09	11.55

$$\frac{{I}_{1}}{{I}_{2}}=\frac{{{\text{g}}}_{1}}{{{\text{g}}}_{2}}\frac{{A}_{1}}{{A}_{2}}\frac{{\lambda }_{2}}{{\lambda }_{1}} {e}^{\left[-(\frac{{E}_{1}-{E}_{2}}{{K}_{B }{T}_{e}})\right]}$$

$$\frac{10000}{20000}=\frac{3}{3}\frac{\left(1.86\times {10}^{6}\right)}{\left(6.4\times {10}^{6}\right)} \frac{\left(696.543\times {10}^{-9}\right)}{\left(794.818\times {10}^{-9}\right)} {e}^{\left[-\left(\frac{13.33-11.55}{1.38 \times {10}^{-23} {T}_{e}}\right)\right]}$$

In accordance with the formula of electron density, the corresponding value was 1 × 10^13^ cm^−3^.

$${N}_{e}\ge 1.6\times {10}^{12}{T}^\frac{1}{2}{\left(\Delta E\right)}^{3}$$

where ${N}_{e}$ (cm^−3^) is the electron density, T is the approximate plasma temperature (0.7178 eV), and $\Delta E$ (following Table 2) is the energy difference between the two states. Based on related reports, for LTE models, electron density must be in the range of 10^19^ – 10^20^ cm^−3^ [52].

It is known that the DBD plasma is non-equilibrium plasma with different temperatures of the plasma species: heavy particles (neutrals and ions) with low temperature, not much higher than room temperature (that is why we use the term of cold plasma) and electrons with the temperature (T_e_ ~ 1–3 eV) of orders of magnitude higher than the temperature of heavy particles.

Following the calculations, the argon plasma recruited in this investigation is classified as a cold one [55]. The cold one is suggested for herbal approaches including germination, root branching, decontamination, etc., as a function of its non-killing effects.

### FGP of treated seeds by DBD

In accordance with similar studies, a research group applied cold plasma to seeds for 30, 60 and 300 s [56]. Another investigation reported the treatment times as 10, 20, 30, 60, 90, 120, 180, 300, and 420 s [57]. Also, a study confirmed that 60, 180, 300, and 420 s exposure times were enough to develop radical species [58]. The longer treatment times, such as 3, 6, 9, 12, and 15 min, were applied on fruits and not seeds due to their potent effects on tropomyosin antigenicity up to 9 min and at the longer times, sulfhydryl proteins were increased, and reduced the amount of the proteins containing α-helix structure [59]. Another group approved that the higher exposure times than 240 s decreased protease activity as a candidate model enzyme in squid. This study's times were 15, 60, 120, 180, 240 and 300 s [60]. Therefore, the treatment times were selected following the literature. Another reason was the development of radical species measured by Optical emission spectroscopy (OES). Radical synthesis was started after 30 s in the corresponding cold plasma system.

**Fig. 6:**
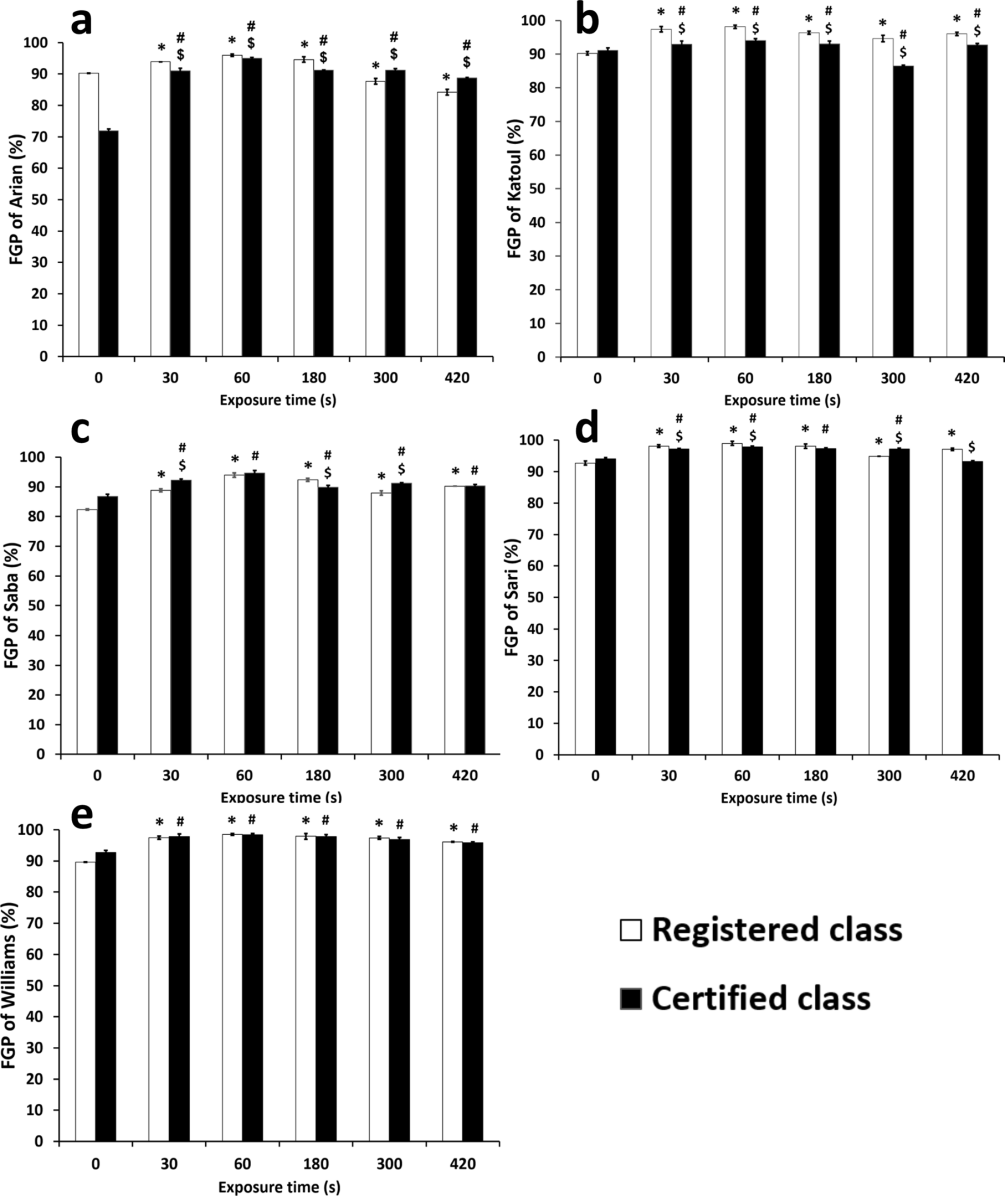
Final germination percentage (FGP) of soybean cultivars and classes. * indicates the significant relation between the registered class of a cultivar as compared to the control group. # indicates the significant relation between the certified class of a cultivar as compared to the control group. $ indicates the significant relation between the registered and certified class of a cultivar within a cultivar. The values lower than 0.05 was considered as significant

The results of the present study are reported in Fig. 6. The FGP values generally ranged between 71.87 ± 0.66 – 98.12 ± 0.61% of the groups. The most considerable difference in seed germination was associated with the certified class of Arian cultivar, compared with the other soybean groups. Regarding the registered type of this cultivar, the plasma time above 300 s significantly reduced FGP (*p*-value < 0.05). Interestingly, increasing the time from 300 to 420 s reduced the FGP variation from—2.625 ± 0.86 to—6 ± 0.84%, respectively. These observations confirmed that the higher plasma exposure time severely decreased FGP (*p*-value < 0.05). The registered class of the Arian cultivar indicates higher FGP than the certified type with exposure times less than 180 s. While with higher time duration, the certified class was exceeded. In contrast, all the registered Katoul seed groups had higher FGPs than the certified group, even at higher plasma processing times (*p*-value < 0.05). Therefore, the highest and lowest FGPs belong to the registered and certified groups with magnitudes of 98.12 ± 0.47 and 86.5 ± 0.25%. In this way, the registered and certified classes started showing negative changes in FGP after 180 and 60 s, respectively. Although there was a significant relationship between all groups of the treated and untreated Saba cultivar (*p*-value < 0.05), FGP reduced with the groups treated for 180 s or longer. The certified group has a higher relative value than the other class (*p*-value < 0.05), unlike the Katoul cultivar. The results were the same for both classes of this cultivar. The Sari and Katoul cultivars’ observations were almost similar, except for the group with a processing time of 300 s. Based on the results of this group, it appears that this group is relatively indifferent to plasma time. Only the certified class with a treatment time of 420 s showed negligible concordance compared to the control group (*p*-value > 0.05). Considering the William cultivar, there was no significant difference between the registered and certified types (*p*-value > 0.05), indicating similarity between their genotypes. Despite this, better germination was observed after the plasma with all exposure time durations. Like the Sari cultivar, this group masked the positive effects of the more prolonged plasma exposure. The highest value of FGP variation of the treated and non-treated groups associated with the certified Arian cultivar was 23.12 ± 0.34%. This value observed with a treatment time of 60 s is quite close to that of a study that reported a 20% increase in soybean germination [61].

### MGT of treated seeds by DBD

**Fig. 7:**
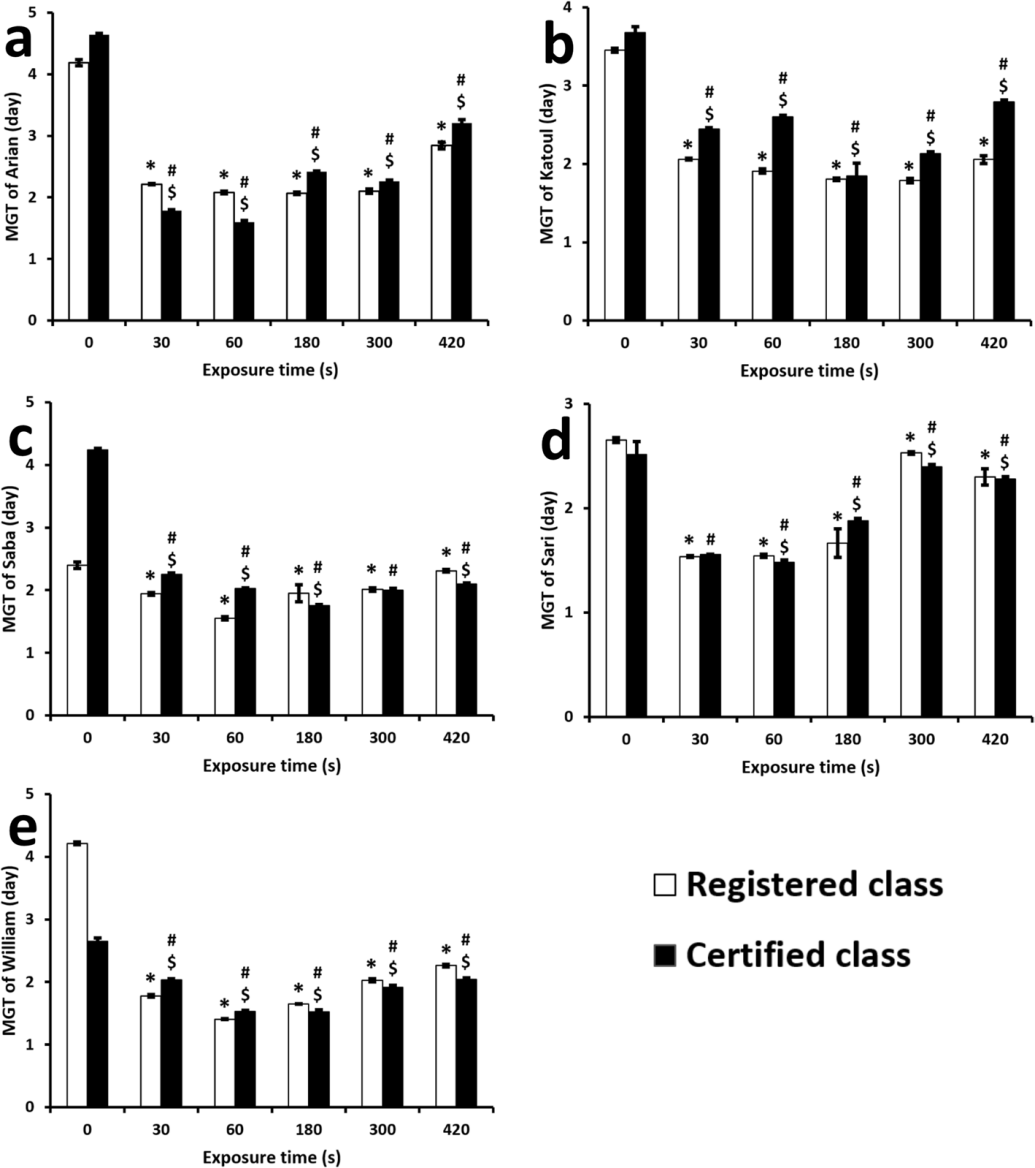
Mean germination time (MGT) of soybean cultivars and classes. * indicates the significant relation between the registered class of a cultivar as compared to the control group. # indicates the significant relation between the certified class of a cultivar as compared to the control group. $ indicates the significant relation between the registered and certified class of a cultivar within a cultivar. The values lower than 0.05 was considered as significant

All plasma-treated groups revealed a significant exposure effect compared with the non-treated group (*p*-value < 0.05). The results have been collected in Fig. 7. For the Arian cultivar, the registered and certified classes behaved differently (*p*-value < 0.05). The lowest MGT values obtained after 60 s were 2.07 ± 0.02 and 1.59 ± 0.02 days for the registered and certified groups, respectively. However, after this time, the value of MGT increases, especially in the certified class. The Katoul cultivar works exclusively, and in this manner, the registered and certified classes indicate the lowest MGT values of 1.78 ± 0.02 and 1.84 ± 0.16 days with the plasma time for 300 and 180 s, respectively. The representative magnitudes of the registered and certified classes of the Sari variety are 1.53 ± 0.008 and 1.47 ± 0.02 days when the plasma time was 60 s.

In contrast, the minimum MGT values related to the registered and certified Saba cultivar were achieved with 60 and 180 s processing times. The corresponding values were 1.55 ± 0.01 and 1.75 ± 0.13 days. The results for the William cultivar were more similar to the Arian, and the group with 60 s indicated 1.40 ± 0.007 and 1.53 ± 0.01 days for its registered and certified classes, respectively. The most substantial effect of the plasma on MGT was observed for the registered class of the William group as 66 ± 0.3%, which was treated for 60 s. In contrast, the weakest positive effect of the plasma on MGT was 30 ± 0.7% for the certified Arian cultivar with a duration of 420 s. Here, the upper and lower times with a relatively lower value of MGT are associated with 300 and 60 s, respectively.

### Root length of treated seeds by DBD

**Fig. 8:**
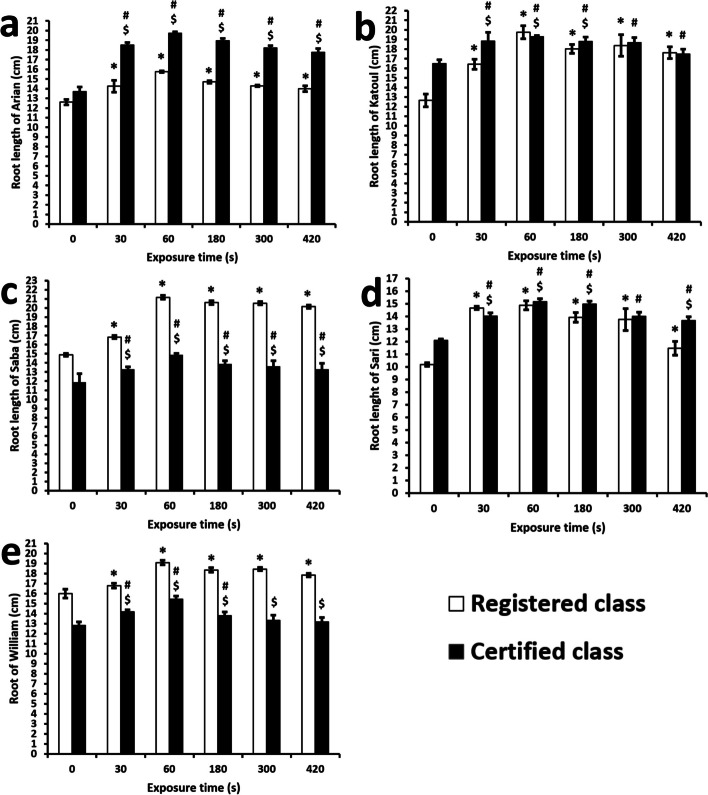
Root length of soybean cultivars and classes. * indicates the significant relation between the registered class of a cultivar as compared to the control group. # indicates the significant relation between the certified class of a cultivar as compared to the control group. $ indicates the significant relation between the registered and certified class of a cultivar within a cultivar. The values lower than 0.05 was considered as significant

In this manner, the values in the present study ranged between 10.20 ± 0.14 cm for the registered class of the Sari cultivar and 21.18 ± 0.21 cm for the registered class of the Saba cultivar (Fig. 8). Accordingly, the minimum and maximum improvement rates (%) were 2.86 ± 0.51% after 420 s and 56.12 ± 2.89 after 60 s. These values are associated with the registered Katoul and certified Williams cultivars. All groups indicate the most extensive length after 60 s. In contrast, the exposure time for 420 s has the lowest value. However, the control group shows the most petite root length, even lower than the value of the treatment time for 420 s.

### Nested factorial analysis of treated seeds by DBD

The results of the analysis of variance are summarized in Table 3. In general, the comparison of the treated and non-treated seeds for their germination properties confirmed the positive effect of this method (*p*-value < 0.05). The considerable variation of the cultivars and classes indicates their incredible genetic diversity. All individual factors, including cultivar, class (within-cultivar), and time were examined along with the combination of cultivar or class with plasma time. The results of these combinations over time supported the critical role of plasma time on FGP, MGT, and root length. Considering plasma time and cultivar or class, it is clear that the respective germination characteristics change at a lower rate. Regarding coefficient variance (%), root length and MGT have the highest and lowest variance, respectively, compared to the other groups.

**Table 3:** Variance evaluation of seed germination properties. DF is abbreviated form of degree of freedom and the values lower than 0.01 was considered as significant and showed by **

Factor	DF	Mean square		
		**FGP (%)**	**MGT (day)**	**Root length (cm)**
**Cultivar**	4	30.1061^**^	56.509^**^	96.105^**^
**Class**	5	21.329^**^	26.60^**^	63.147^**^
**Time**	5	46.790^**^	50.306^**^	40.72^**^
**Cultivar × Time**	20	98.61^**^	99.24^**^	36.3^**^
**Class × Time**	25	28.94^**^	77.39^**^	64.4^**^
**Error**	180	70.17	49.11	72.0
**Coefficient of variation (%)**	-	3.74	63.3	37.5

### Pearson correlation of FGP and root length

Based on other reports there is a closed correlation between the root length and seed germination [62]. Following other observations, root length is a crucial trait to describe seedling vigor [63]. The mechanism has been reported that a higher amount of hormones in seedlings triggers more remarkable growth of roots [64]. Another investigation confirmed the direct relation between the lower root density and other herbal characteristics on the healthy condition of seedlings [65]. Some studies cited the same fate of root length and seed growth after toxic materials treatments. In this manner, a group reported that after the incubation of seeds with Ni, both root length and seed growth were inhibited [66]. Therefore, a close dependency can be considered between the root length and the healthy level of seedlings. In accordance with Table 4, the coefficients of correlation among the FGP and root length were significant in both classes of Saba cultivar, the certified class of Arian and registered of Katoul (*p*-value < 0.05). In contrast, the others indicated insignificant relationships (*p*-value > 0.05). However, the correlation coefficient values were positive in all groups, approving positive relations between the germination and root length of the seeds. If the sample size were enlarged, at higher FGP values, longer roots could be expected. Regarding this phenomenon, the germination percentages of the seeds may be combined to examine the length of their roots. Therefore, a strategy that can induce germination may be capable of enforcing root growth.

**Table 4:** The correlations between FGP and root length with the seed number of 6. ** presents the p-values lower than 0.01, and * presents the *p*-values lower than 0.05

FGP (%) \ Root length (cm)		Correlation coefficient (r)	*p*-value
**Cultivar**	**Class**		
Sari	Registered	0.760	0.079
	Certified	0.742	0.091
Saba	Registered	0.838*	0.037
	Certified	0.887*	0.018
Arian	Registered	0.509	0.302
	Certified	0.992**	0.01
Katoul	Registered	0.840*	0.036
	Certified	0.133	0.802
Williams	Registered	0.799	0.056

### Electrical conductivity of solutions containing treated seeds by DBD

**Fig. 9:**
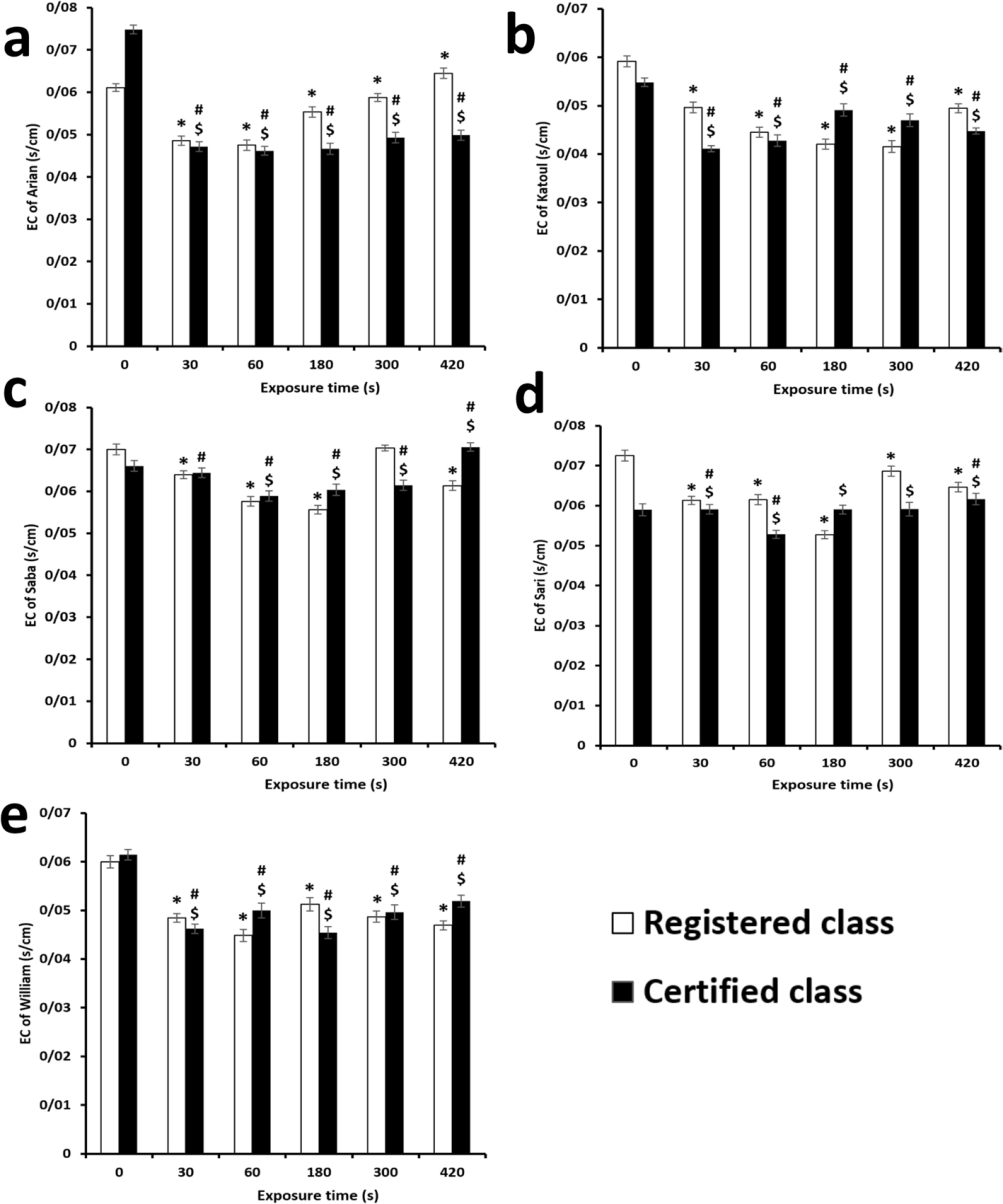
Electrical conductivity (EC) of soybean cultivars and classes. * indicates the significant relation between the registered class of a cultivar as compared to the control group. # indicates the significant relation between the certified class of a cultivar as compared to the control group. $ indicates the significant relation between the registered and certified class of a cultivar within a cultivar. The values lower than 0.05 was considered as significant

The registered Arian cultivar was recorded to possess 0.048 ± 0.001 s/cm with the seeds treated for 30 s, while this value increased to 0.064 ± 0.001 s/cm with an exposure time of 420 s (Fig. 9). These time treatments are the minimum and maximum values of the electrical conductivities of the Arian variety. In this manner, the certified one shows 0.047 ± 0.001 and 0.049 ± 0.001 s/cm for 30 and 420 s exposure times, respectively. The rate of increase in conductivity is different between the classes approving their varying tolerances to plasma irritation based on their various genotypes. The behavior of the two classes of the Katoul cultivar is reversed. The certified one illustrated 0.041 ± 0.0006 and 0.049 ± 0.001 s/cm for the groups treated for 30 and 180 s, respectively. Herein, the electrical conductivity reduces to 0.044 ± 0.0006 s/cm as the discharge time increases to 420 s. Significant fluctuations in the electrical conductivity of the Saba cultivar resulted, and the value of registered and certified classes changed to 0.07 ± 0.0006 and 0.07 ± 0.001 s/cm after the plasma treatments for 300 and 420 s, respectively. Notably, the relationship between the treated and untreated registered classes was insignificant (*p*-value > 0.05). However, the registered group showed a lower value of 0.061 ± 0.001 s/cm after 420 s. The same is repeated for the Sari cultivar, including registered and certified types. The electrical conductivity of the registered one reduced with the group treated for 420 s, while the certified group had a positive slope in terms of treatment time. The certified class treated for 180 and 300 s showed an insignificant difference from the control (*p*-value > 0.05). Nevertheless, after the treatment for 420 s, the conductivity increased considerably to 0.062 ± 0.001 s/cm (*p*-value < 0.05). For the registered Williams cultivar, the value reduced after 300 s to 0.048 ± 0.001 s/cm, while the certified group had an increasing trend at higher treatment times, although there was some fluctuation. The certified group is generally more uniform in terms of conductivity than the registered group. The electrical conductivity was triggered with more prolonged plasma treatment for all soybean groups. Herein, most plasma-treated seeds indicated lower electrical conductivity than the control group.

### Contamination of treated seeds by DBD

**Fig. 10:**
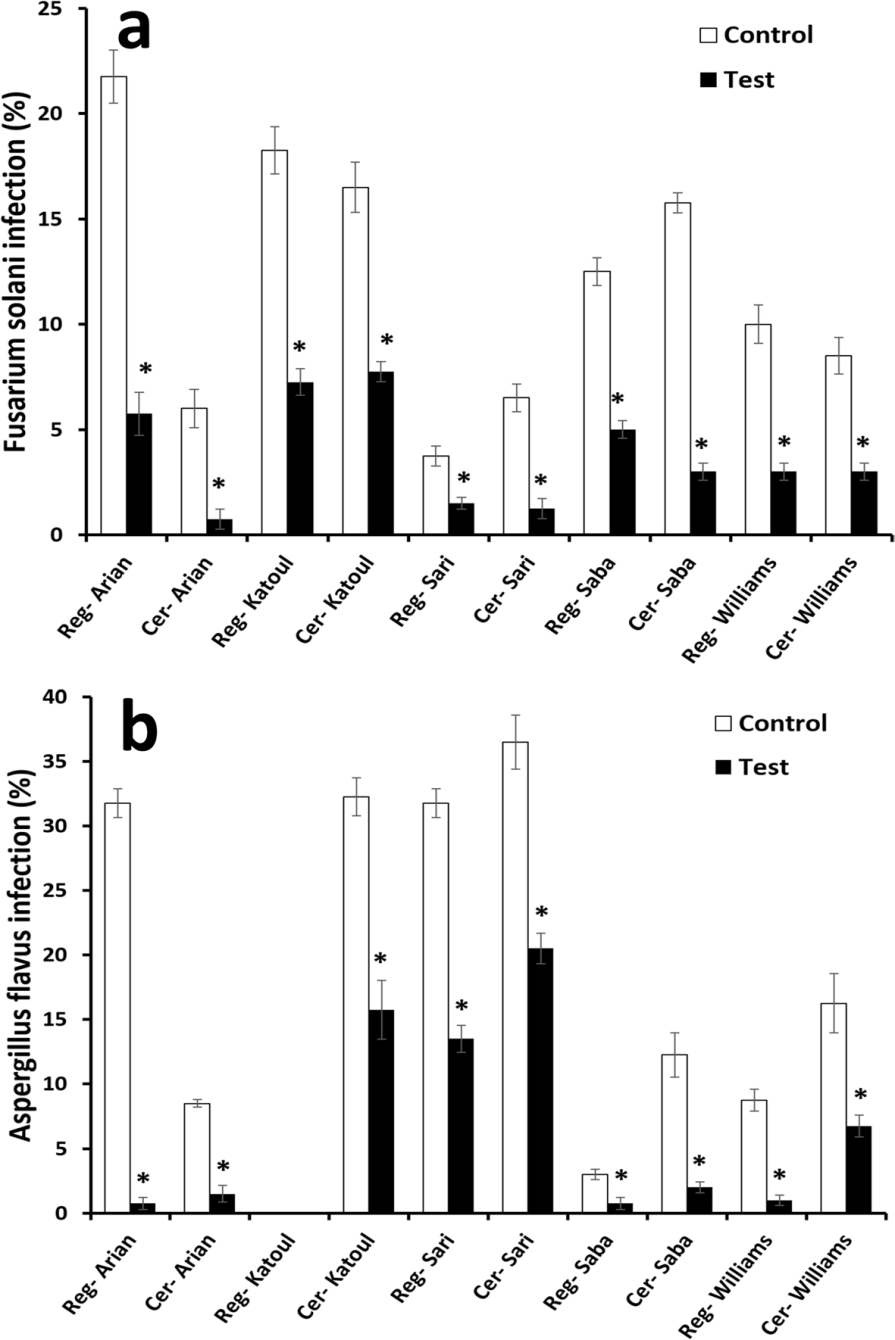
Microorganism infection of soybean cultivars and classes. * indicates the significant relation between the registered class of a cultivar as compared to the control group. # indicates the significant relation between the certified class of a cultivar as compared to the control group. $ indicates the significant relation between the registered and certified class of a cultivar within a cultivar. The values lower than 0.05 was considered as significant. Reg and cer are abbreviated forms of registered and certified classes. The control and test are the seeds without and with plasma treatment

Fungal infection was evaluated by seed examination for the species, including *Fusarium solani* (F. solani) and *Aspergillus flavus* (A.flavus) species. The former is known to infect most crops [67] by invading specifically the roots of beans [68]. The latter is dominantly able to induce aflatoxins in plants [69]. Here, the plasma exposure time for this assessment is assumed to be 180 s based on the electrical conductivity values discussed above. The fungal infection results are collected in Fig. 10. The certified class seems more resistant than the registered one for the Arian variety because it is less susceptible to infections with the respective fungi. Despite this, there was a significant relationship between this group’s treated and non-treated versions (*p*-value < 0.05). While regarding F. solani, the undischarged Katoul cultivar indicated 18.25 ± 1.11 and 16.5 ± 1.19% for the registered and certified classes, respectively. The values were reduced to 7.25 ± 0.63 and 7.75 ± 0.48% depending on the sterilizing activity of the plasma. Only the certified class of this cultivar was infected by A.flavus around 32.25 ± 1.49%, which decreased after the exposure to 15.75 ± 2.29%. The reduction rates of the registered and certified classes were 60 ± 3.9% and 80 ± 2.6% for F.solani. In contrast, the certified control indicated a lower A.flavus infection of 43 ± 4.3% compared with the registered group of 57 ± 6.3%. In a similar process for the Saba cultivar, the reduction in A.flavus contamination was greater than 50% for the treated seeds. In this manner, the prevalence of F.solani infection for the registered class was reduced from 12.5 ± 0.65% to 5 ± 0.41% (p-value < 0.05). On the other hand, the value of 15.75 ± 0.48% for the certified Saba variety was decreased to 3 ± 0.41% after plasma irradiation for 180 s. For A.flavus strain, the registered group indicated 3 ± 0.41 and 0.75 ± 0.48% for the non-treated and treated samples. Successful sterilization by the plasma occurred for the Williams cultivar. The registered seeds confirmed 10 ± 0.91% lessened after the plasma to 3 ± 0.41%. In the same behavior, the certified class had 8.5 ± 0.87 and 3 ± 0.41% for the untreated and treated groups. According to the significant relationship between the treated and non-treated groups, it could be concluded that the plasma can indeed inactivate the seeds and ensure their health. Overall, the contamination removal rates (%) associated with F.solani and A.flavus were 67 ± 4 and 65 ± 3.1%, respectively. These values were insignificant (*p*-value = 0.5), confirming the effect of cold plasma on decontamination, which was relatively similar for both fungal strains (Supplementary information files: S1, S2, S3, S4 and S5).

## Discussion

Higher seed germination rate, seedling growth, and length are some of the biological functions that suggest a positive role of cold plasma [70]. Moreover, the coating of the seeds is subject to the action of plasma components, including electrons, ions, and reactive species. This layer is peeled off, and free radicals can penetrate inside the seed. This occurrence can potentially induce the noted metabolic functions of seeds [71]. Cold plasma needs to be optimized at voltage, power, exposure time and distance of seed and plasma tip to induce a higher level of seed germination. Due to the damaging impact of cold plasma on seed morphology at strong contacts, surface ablation may happen, and healthy biological components will be injured. Thus, it is easy to change exposure times to discover an optimum treatment time. Also, at longer cold plasma treatment, the water uptake by seeds reduces, and the moisture evaporates quickly, damaging the seed structure [72]. It has been approved that longer exposure times of cold plasma reduce pH values due to higher penetration of nitrites and nitrates to inside seeds. This high acidity retards germination due to the negative impact of high nitrogen compounds on germination [73]. However, the tolerance of various seed types is different and confirmed a close correlation between the plants' tolerance and their polyphenol concentration [74]. In this manner, the primary killing process of biomolecules, including lipids, proteins and DNAs, peroxidation usually happens at longer plasma treatments [55]. Low germination at longer treatments is generally related to high input power, which makes much more electron density, and the cold plasma behaves similarly to UV radiation [75]. Therefore, the heat at the seed surface is increased, and its biological functions are damaged or retarded. Overall, the extended exposure time is interesting when microbial inactivation of seeds has been considered rather than germination approaches. In this way, the seeds intended for storage or feeding benefit from cold plasma more. In contrast, shorter treatment times must be considered for cultivating seeds.

The corresponding reactive species have been recognized by OES, confirming the possibility of argon plasma activating bioactive compounds [76]. Regarding the decontamination mechanism of hydroxyl radicals, the destruction of microbial cellular membranes has been reported via lipid oxidation by these chemical groups. Indeed, the radicals subtract H atoms from unsaturated carbon bonds and form malondialdehyde (MDA) due to oxidation reaction [55, 77]. The radical nitrogen species (RNS), including N2 I and N2 II types, inactivate bacteria and their spores by destroying proteins, lipids and nucleic acids [78]. Another mechanism relevant to the decontamination potency of nitrogen radicals has been confirmed due to their ability to damage surface polysaccharides [10]. After disrupting microbial membranes, intracellular components are leaked out [79]. A study approved that the main antimicrobial reaction of radicals is related to oxygen radical species (ROS), including atomic oxygen (O), metastable oxygen (O2*), superoxide (O2 −), ozone (O3), hydroxyl radical (OH −) and hydrogen peroxide (H2O2). The main ROS among the corresponding chemical groups is Ozone (O3), atomic oxygen (O) and hydroxyl radical (OH∙) [80]. The mechanism has been reported morphological alterations, membrane peroxidation, intracellular content leakage, protein oxidation and DNA breakage [55]. However, atomic oxygen cannot rupture the membrane, and its role depends on the oxidation of internal oxidation [81]. Similarly, argon radicals make sterilization by destroying cytoplasmic walls and cellular organelles such as DNA, proteins and carbohydrates [82]. Furthermore, seed growth can be promoted after cold plasma exposure. The mechanism is related to some extent to the type of radical species. Radicals generally etch the seed surface, and nitrate can enter the seeds [83]. Another mechanism is the higher wettability of seeds after their treatment with radicals [55]. This lower water contact angle increases water uptake by seeds. Based on Fridman, radicals can damage DNA and stimulate signals such as the synthesis of growth factors. These factors change seed fate via the breakdown of the dormant stage and, finally, a higher rate of germination [84]. The surface erosion and higher water absorption disturb the seeds' internal space's osmotic pressure; accordingly, the seed increases osmotic-adjustment components such as sugar and proteins. The higher concentrations of these metabolites act as a starting signal of germination [85]. In a similar mechanism, the level of gibberellin is increased to break down nutrients stored in seed endosperm due to the requirement for more metabolites [86]. Also, RNS molecules lead to the acidification of water surrounding seeds (pH < 7), and this condition causes chapping of the waxy layer in the seed cover. Afterward, the seeds can absorb water and nutrients, promoting higher germination [87]. The N-fixation of seeds due to plasma lighting has explained a mechanism of cold plasma on seed germination. In this phenomenon, the lighting energy converts N2 into nitric oxide, nitrite, nitrate, dinitrogen trioxide, dinitrogen pentoxide and ammonia. These nitrogen fertilizers provide a source of nitrogen for the plant and make a higher rate of germination [88, 89]. The ROS molecules, particularly hydrogen peroxide, superoxide and singlet oxygen, degrade metabolites via their oxidation. On the other hand, they increase the expression of antioxidant enzymes, enhancing the resistance of seeds against drought stress and seed growth [90]. Similarly, the MDA amount was reduced after plasma exposure in tomato germination due to the inhibitory role of these enzymes on lipid peroxidation [91].

In a related examination, a mixture of oxygen and argon as the plasma carrier gas was applied to rice seeds, and the results approved longer roots and bodies due to their higher physiological metabolites [92]. In this regard, an influential role of argon plasma after 5 min on the seeds of *Ocimum* *basilicum* was observed to increase germination by 5 times compared with the control group. In this study, the power value was not noted, but the voltage and frequency values were 35 kV and 30 kHz [93]. Another research approved the release of seed dormancy in wheat as a function of nitrogen radicals in the presence of argon as the plasma carrier gas [94]. As previously documented, cold plasma triggers treated seeds’ germination and other physiological activities. At the same time, argon gas was used by a group to evaluate the germination of mustard seeds (*Brassica nigra*), and related observations confirmed improved FGP values for up to 3 min. The texture of the treated seeds was coarser than that of the non-treated group. This study's power, voltage, and frequency were 7.3 W, 11.7 kV, and 50 Hz, respectively [95]. The present study's values were 100 w, 5 kV, and 8 kHz. Another investigation used the same plasma gas for radish (*Raphanus sativus*) and carrot (*Daucus carota sativus* *L.*) seeds. The assessments confirmed the plasma for 3 min enhanced seed germination when the power, voltage, and frequency values were 17.9 W, 11.32 kV, and 50 Hz [96]. Plasma irradiation using N_2_ (79%) and O_2_ (21%) on two cultivars of *Oryza sativa* for 1 min increased photosynthetic hyperpigmentation, chlorophyll fluorescence, and antioxidant enzyme activity. The voltage and frequency were 10 kV and 2 kHz, and the power value was not noted [97]. In one study, the plasma generated by air, even for up to 7 min, produced a higher germination rate of sunflower seeds (*Helianthus annus* *L.*), and abscisic acid content, as a dormancy marker, was reduced. The power, voltage, and frequency values were 0.35 W/cm^3^, 12.7 kV/m, and 5.28 MHz [13]. These various treatment times may be related to different configurations of the DBD device, including frequency, voltage and part size. Lower FGP of the registered type of Arian cultivar is associated with more surface deterioration with a plasma exposure time of more than 300 s. Consequently, the seed loses more significant water and nutrients. As a result, the structure of the seed is compacted and eventually, germination is delayed [95].

In contrast, the registered Arian group had a higher FGP than the certified one when the treatment time was less than 180 s. While with a higher duration, the certified class was exceeded. To our knowledge, these various observations may be related to their different genotypes and stress tolerance. The corresponding group showed higher values when the plasma time was increased to 300 and 420 s. For FGP with a more prolonged plasma exposure, the Katoul cultivar contrasted with the Arian group. This result agrees with an examination that introduced the treatment time of 60 s as the critical point and approves that the lethal effects of cold plasma on seeds become more potent as the time increases. The corresponding time was recorded when the power was 50% of 1100 W [98]. A maximum FGP of 23.12 ± 0.34% related to the certified Arian variety resulted in an exposure time of 60 s for this group. This value is quite close to that of a study reported to improve the germination rate of soybeans by 20%. However, the mentioned evaluation's power, voltage, and frequency were 400 W, 20 kV, and 14 kHz, respectively [61]. On the other hand, cold plasma, via increasing seed vigor and physiological activity, can change the MGT value to a lower amount than the non-irradiated groups [99]. Its mechanism involves the interaction of the plasma with the seed surface as a substrate for radiation, heat conduction, and, finally, the generation of chemically active compounds [100]. Consistent with the plasma-activated water (PAW) tests, no differences existed between the treated and non-treated groups [101, 102]. These observations suggest that if cold plasma is directly applied to seeds, results regarding their germination percentage or time, physiological functions, and decontamination capabilities would be evident. Oppositely, longer plasma durations, such as 420 s, had a minimal impact of 30 ± 0.7% on MGT, as seen for the certified Arian cultivar. The corresponding result is consistent with the adverse effect of cold plasma on seeds that have been discharged for a longer time. With longer plasma treatments, the temperature of cold plasma is converted to a higher degree that evaporates volatile oils in seeds [103]. In agreement with the results of the present study, the maximum time to a lower value of MGT was 4 min for radish (*Raphanus sativus*) and carrot (*Daucus carota sativus L.*) seeds. The parameter values of the cold plasma in this study were 17.9 W, 11.32 kV, and 50 Hz for power, voltage, and frequency [96]. Root length is another physiological property of plants positively influenced by cold plasma [79]. Here, all treated groups revealed more extended root sizes, even when the time was increased to 420 s. The highest enhanced root length of soybean (*Glycine max L. Merr cv. Zhongdou 40*) with non-thermal air plasma was 21.95%. The optimized plasma time to achieve this positive effect was 15 s when the power and frequency were 80 W and 13.56 MHz [5]. The considerable difference between this examination and the present study is related to their respective plasma frequencies of 13.56 and 8 kHz. Another group confirmed longer root lengths of 1884.7 mm in wheat with argon gas compared to their air control group. Its improvement rate was 7.12% [104]. Such low values can be related to their minimal frequency of 50 Hz. However, their frequency was 13 kV, which is higher than the value of the present study (5 kV). Regarding the effect of the applied voltage, a group demonstrated that when the voltage was 11 kV, a treatment time of 4 min was sufficient to achieve the highest root length, such as 2094.0 mm of wheat seed. The gas employed was air at atmospheric pressure, and the frequency was 50 Hz. The value of power was not noted [105]. A similar evaluation was done for wheat seeds. Their observations indicated that the highest enhanced root length was obtained after 7 min in air plasma with frequency, voltage, and power of 50 Hz, 13 kV, and 1.5 W [106]. Comparing the plasma discharged times of 60 and 180 s with the voltage of 5 kV in the present study shows that this inconsistency is related to their different frequencies and seed origins. Different characters among seed varieties must be their genetic and geographical variations [107]. In this manner, the tolerance of seed strains against different stress, such as drought, heat [108], osmotic [109], chilling [110], a combination of stressful conditions [111] etc., are dependent on their physiological and biochemical aspects. These variations originate from their specific metabolism, antioxidative defense, respiration rate, lipid peroxidation, enzyme synthesis, carbohydrate content, morphological properties such as the thickness of seed coating (bare grain layer compared to thick one), amount of proanthocyanidins [112] and hormonal levels. If we consider cold plasma a stressful situation, setting the parameters belonging to this method for any seed strain would be needed. However, some studies confirmed insignificant differences between varieties of seed germination [113]. An investigation confirmed that the drought-sensitive oilseed rape had a higher germination rate after cold plasma treatment than the tolerant type. Despite this, both varieties indicated longer root and shoot in the treated groups [90]. Similar observations proved that the cold plasma results vary between rice [114] and wheat [115]. It has been found that dicotyledonous plants are more sensitive to plasma than monocotyledonous types. This phenomenon is related to their different enzyme activities not number of enzyme types [116]. Therefore, the optimization of cold plasma conditions has been suggested for a certain cultivar.

If the dormancy phase of a plant is considered, different crops indicate various durations and cycles of dormancy, approving their specific biological potency for germination or root and seedling growth. Therefore, cold plasma results strongly correlate with their ability to sprout. However, it is not deniable that there are many similarities among herbal classes. Mainly, cold plasma promotes germination via modifications of a plant's biochemical and molecular properties to a level with a higher chance of giving up the dormancy phase or higher physiological activities [117]. These differences make distinguishable behavior of various plants after cold plasma, and each herbal strain needs an optimized set of cold plasma, including voltage, frequency, time and even dimensions of apparatus for their germination, root branching, antioxidant enzymes, photosynthetic pigments, phenol content, etc. [118]. Following the genotype of plants, their tolerance against salt, osmotic, thermal and moisture stresses are different. Even for better consequences, this method should be combined with other techniques, such as physical or chemical types in some cultivars. While in other cultivars, cold plasma can trigger seed growth solely. In this manner, the expression level of an enzyme, oligosaccharides amount or other membrane components, signaling pathway of resistance against pathological microorganisms and morphological properties such as cracks on seed coating determine the sensitivity degree of a plant strain to cold plasma. For example, when respiratory burst oxidase homologue 1 (RBOH1) was silenced, the cold stress tolerance has not happened. In contrast, in typical situations, cold plasma can upregulate this gene and the expression of ABA happens in the following [119]. Besides intraspecies differences, seed macroscopically properties such as size, shape, color, the structure of internal components and water amount can affect the observations. These particular traits originate from their genetic polymorphism. In this manner, the same lot and variety seeds are polymorphic due to their different dormancy degree and other physiological differences [120]. However, the interactions between internal and external properties in seeds are complicated, mainly when they are considered simultaneously with parameters of cold plasma, such as its treatment times [121]. Similarly, tomato seedlings indicated lower oxidative damages after cold plasma despite its high phytohormone synthesis and expression of pathogen resistance genes [122]. At the same time, this oxidation may be helpful to create crakes on seed coating.

Non-thermal plasma increases cell membrane permeability; thus, some intracellular biomolecules can leak out [123]. The compounds which are released, improve the electrical conductivity of the seed-soaking water. In this manner, the certified Arian cultivar showed 0.046 ± 0.001 and 0.05 ± 0.001 s/cm for 60 and 420 s exposure times, respectively. These different conductivities between the classes approve the relationship between their tolerance to plasma and genotypes. The behavior of the two classes of the Katoul cultivar is reversed. Therefore, the certified one illustrated 0.041 ± 0.0006 and 0.049 ± 0.001 s/cm for the groups treated for 30 and 180 s. The electrical conductivity has dropped to 0.044 ± 0.0006 s/cm when discharged time is increased to 420 s.

According to other references, the value must be increased in the treated group compared to the control (non-treated group). In contrast, in our study, the electrical conductivity of the treated groups was lower than the control. However, in the certified group of Saba cultivar, the value was increased in the treated group for 420 s. Moreover, the high electrical conductivities in most cases increased when the treatment times were longer. However, the higher amount of electrical conductivity in the control group against the experimental types confirmed no morphological changes in seeds with the cold plasma in the present study. Another reason could be related to the destruction of biomolecules due to extended cold plasma. Also, this lower value may be related to the initiation of seed dormancy at more prolonged periods of cold plasma.

In other words, seed induction has a critical exposure time, and exceeding this value could result in adverse effects. Another justification is the correlation between electrical conductivity and the lifetime of free radicals. Thereby, the activity of these radicals is eliminated before they significantly impact seed permeability [124]. Due to high value of electrical conductivity at higher and stronger plasma treatments, we can conclude that this property is related to the degree of plasma injuries on seeds. Therefore, at higher values, the parameters of cold plasma should be optimized to reduce the level of seed damage. Some studies reported an exact application of electrical conductivity. For example, a group introduced this parameter as a degree of chilling injury in cotton seeds [125]. In this manner, when the time of cold plasma is enhanced by the aim of more robust seed germination and decontamination potency, the value of electrical conductivity increases. Thus, this property could detect damage to the depth of seed coating and confirm the maximum level of cold plasma. In other words, a high electrical conductivity value is not interesting for all cases due to its harmful impacts as a function of a high power and longer treatment of cold plasma.

The seeds treated by cold plasma release biomolecules when their surface tissue is damaged; the same goes for bacteria and fungi when seeds are discharged for sterilization. On the other hand, exposure to shorter durations is more interesting than medium or high periods [126]. Thus, an exposure time of 180 s was chosen to evaluate the decontamination ability of cold argon plasma in the present study. At first glance, the different decontamination efficacy between the two fungi confirmed that the microorganism strain could affect the antifungal activity of the plasma. Therefore, the plasma parameters should be adjusted when the target contamination agent differs [127]. On the other hand, when comparing the contamination removal rates of the two strains, a relatively similar value confirms the same effect of the cold argon plasma on the corresponding strains. Overall, consistent with significant relations between the treated and non-treated groups, it can be concluded that the plasma indeed inactivates the microorganisms of the seeds and guarantees their health. The fungicidal mechanism of cold plasma is similar to UV radiation. In this regard, the seed surface is bombarded by atomic oxygen and radicals [128]. Based on this process, spore walls are damaged or ruptured [10], and biomolecules such as proteins, lipids and DNAs start oxidizing or dissecting. Microbial decontamination includes microorganism types of viruses, bacteria, fungi and prions. In the present study, the fungi group is considered. Similar mechanisms are involved regarding the different decontamination potency of cold plasma on various fungal strains. However, as well as seed varieties, fungal strains possess specific genotypic origins determining their pathogenic nature, morphology and resistance against sterilization methods. The base of different reactions in fungi against sterilization methods, such as cold plasma, is related to their DNA diversity. In fact, despite some identical DNA sequences, they have significant differences in structure and composition. The hydrophobic degree of fungi spores' surface is a factor that manipulates the sufficiency of cold plasma on sterilization outcomes. In this manner, hydrophobic spores easily create aggregates in contrast to singular distributions of hydrophilic ones. Therefore, the hydrophilic seed is more vulnerable to cold plasma due to electroporation formation in its walls [129]. Different surface compositions of fungi need particular cold plasma powers based on their distinguishable thermodynamic basis. Peng Sun et al. investigated the decontamination of cold plasma against Candida albicans, Candida krusei, and Candida glabrata. The observations confirmed that Candida glabrata indicated 100% sterilization in 2 min, Candida krusei showed 91% after 10 min, and Candida albicans possessed 93% after 10 min [130]. Another group reported that to reach the maximum fungicidal effect of cold plasma for Fusarium, Alternaria and Stemphilium species, the treatment time should be 10–15 min. However, in some cases, their inhibition percentages were less than 5% [128]. Selcuk et al. exhibited by examination of Aspergillus spp. and Penicillum spp. that the fatal outcomes of cold plasma correlates with plasma gas, plasma time, and seed surface properties [10].

The applicability of cold plasma could be discussed via different aspects. Current methods to induce germination or decontaminate seeds are classified into physical and chemical types. Irrespective of their harmful effects on humans and the environment, each method is not economically attractive. However, mass production of cold plasma still accompanies significant problems such as the undesired and unpredicted results in actual farming practices, the high cost of vacuum conditions, or usage of a particular gas. On the other hand, seed varieties behave somewhat differently when treated with cold plasma. All corresponding reasons lead to setting special conditions for cold plasma type and seed species. Therefore, despite many references introducing this method as a cost-effective technique [131–133], its high lipid oxidation, changing color and morphology of fruits explain that this method should be selected conservatively for the noted applications. It seems that the scalability of cold plasma due to its operational feasibility would be applicable, and based on involved research, large-scale practices of this method have been suggested. It seems that the localization of the plasma plume at an electrode tip limits the scalability opportunities of this method [134]. Thus, a flexible electrode system is needed to generate radicals, and this electrode should homogenously work when the device's size is enlarged. Printed, knitted or hybrid electrodes have been suggested to be considered when cold plasma is aimed to scale up [135]. High gas consumption in cold plasma is another concern for large-scale works of cold plasma, and regarding this, the presence of a gas flow is another requirement. In this manner, the system should be modified to replace expensive gases like argon with atmospheric air or other gases [136]. A study fabricated a similar apparatus to cold plasma, namely low-pressure microwave plasmas, to sterilize foods. They confirmed its favorable industrial applications [137]. Another drawback that should be considered for industrial applications is the contamination of samples with the electrodes of cold plasma during the treatment process. A reliable replacement of these electrodes could solve this problem or the employment of contactless electrodes [138]. As a whole, scalability and practical implementations of this system may be possible, although some considerations must be applied. A non-pathogenic process is needed to obtain safe industries. For this approach, the factors including no considerable manipulation of seed or micro-organism genotypes and eco-friendliness should be considered. For this aim, plasma chemical composition, radical density and plasma time must be carefully selected, evaluated and controlled [26]. Also, the damage to microorganisms in decontamination must be examined to detect the possible mutations in their DNA. This strategy may inhibit undesirable effects and help to reach an ideal sterilization setup. On the other hand, under the regulations of the Agricultural Marketing Service (USDA-AMS), the quality of the final fruit must be approved to prevent future human health issues [139]. On the one hand, cold plasma functions immediately after turning on the system. Additionally, the developed radical species are unstable; thus, the unpredicted reactions will be minimal. Therefore, it would be concluded that this method is a technology without significant pollution and has been classified as an advantageous green process [140] for seed germination or decontamination. Despite this, the long-term outcomes of this method on microorganisms, plants and humans should be evaluated, and it is clear that these examinations limit its industrial scaling. At least, it is an emergency to consider some limitations of the implementations of cold plasma in industry. All well-known seed germination and sterilization methods are magnetic exposure, genetic recombination, other light sources such as UV, thermal shocking process and chemical fertilizers. Nowadays, due to their high cost and pollution, most of these techniques are discarded and replaced with newer systems, such as cold plasma. If magnetic treatment is compared to cold plasma, this method is weak and, in most cases, cannot induce germination [141]. In some studies, magnetic field and cold plasma were combined. In this manner, the plasma without a magnetic field indicated higher seed germination and lower fungal resistance. In contrast, lower germination and higher microorganism tolerance were detected in the synergistic model of the plasma and magnetic field [142]. Other listed methods, such as genotype modification, UV and hot water incubation, are often accompanied by many unpredicted damages and lower potency. However, radical species in cold plasma may cause breakdowns in DNA strands, although these problems are seen in most methods, especially when this method has been combined by other techniques such as UV [143]. Environmental pollution and some human diseases due to allergic reactions to chemical methods such as fertilizers [26] highlight the considerable potency of cold plasma over other processes.

## Conclusions

Cold argon plasma triggers seeds’ physiological activities and protects them from microbial infection. Soybeans, the main crop in the food industry, may be of lower quality due to harsh environmental conditions. Not only the FGP values of the corresponding seeds increased to 23.12 ± 0.34%, but the plasma efficiency on MGT was 66 ± 0.3%. These satisfying values indicate its considerable strength. Moreover, the most incredible root length percentage was 56.12 ± 2.89%, and all these seed properties were achieved by plasma treatment in just 60 s. However, these characteristics vary between the cultivars and within cultivars (classes).

In contrast, electrical conductivity only increased after a treatment period of 180 s or longer. Therefore, the corresponding plasma duration was used to detect seed contamination with A.flavus and F.solani. The observations confirmed that despite the crucial effect of the plasma in decontamination, both microorganisms were equally pathogenic between the cultivars and their classes. Subsequently, cold argon plasma is recommended for seeds susceptible to adverse environmental conditions. The employed system was designed to survey in lab-scale experimental, and for large-scale farming, the system must be changed. Precisely for this application, the authors of this article tried to investigate at the experimental level, and then the application will be progressed to actual farming practices.

### Supplementary Information

**Additional file 1: Supplementary data of Table 2.** ANOVA for seed germination and seedling growth characteristics in soybean (a) Germination percentage (b) Germination Rate (c) Germination index (d) Root length (e) Seedling length (f) Seedling dry weight.

**Additional file 2: Supplementary data of  Table 3.** Effect of cold plasma on seed germination properties in soybean for Germination index.

**Additional file 3: Supplementary data of  Table 4. **Effect of cold plasma on seed germination properties in soybean for Root length (cm).

**Additional file 4: Table 5.** ANOVA for Antioxidant enzymes activity (a) catalase enzyme (CAT), (b) superoxide dismutase (SOD), (c) ascorbate peroxidase (APX). 

**Additional file 5**: **Supplementary data of  Table 6.** Effect of cold plasma on seed germination properties in soybean for APX activity  (μM g-1 FW min-1).

## Data Availability

All data generated or analysed during this study are included in this published article (supplementary information files: S1, S2, S3, S4 and S5).

## References

[1] Ganesan AR (2021). Application of cold plasma on food matrices: a review on current and future prospects. J Food Process Preserv.

[2] Tabares FL, Junkar I (2021). Cold plasma systems and their application in surface treatments for medicine. Molecules.

[3] Michael AL, Allan JL (2005). Principles of plasma discharges and materials processing.

[4] Tong J (2014). Effects of atmospheric pressure air plasma pretreatment on the seed germination and early growth of andrographis paniculata. Plasma Sci Technol.

[5] Ling L (2014). Effects of cold plasma treatment on seed germination and seedling growth of soybean. Sci Rep.

[6] Menashi W (1968). Treatment of surfaces. Sl patent No. 3383163.

[7] Thirumdas R, Sarangapani C, Annapure US (2015). Cold plasma: a novel non-thermal technology for food processing. Food Biophys.

[8] Misra N, Jo C (2017). Applications of cold plasma technology for microbiological safety in meat industry. Trends Food Sci Technol.

[9] Mahnot NK (2020). In-package cold plasma decontamination of fresh-cut carrots: Microbial and quality aspects. J Phys D.

[10] Selcuk M, Oksuz L, Basaran P (2008). Decontamination of grains and legumes infected with aspergillus spp. and Penicillum spp. by cold plasma treatment. Bioresour Technol.

[11] Park H, Puligundla P, Mok C (2020). Cold plasma decontamination of brown rice grains: impact on biochemical and sensory qualities of their corresponding seedlings and aqueous tea infusions. LWT.

[12] Jiang J (2014). Effect of seed treatment by cold plasma on the resistance of tomato to Ralstonia solanacearum (bacterial wilt). PLoS ONE.

[13] Mildažienė V (2019). Treatment of common sunflower (Helianthus Annus L.) seeds with radio-frequency electromagnetic field and cold plasma induces changes in seed phytohormone balance, seedling development and leaf protein expression. Sci Rep.

[14] Ivankov A (2021). Changes in agricultural performance of common buckwheat induced by seed treatment with cold plasma and electromagnetic field. Appl Sci.

[15] Bormashenko E (2015). Progress in understanding wetting transitions on rough surfaces. Adv Colloid Interface Sci.

[16] Sera B (2010). Influence of plasma treatment on wheat and oat germination and early growth. IEEE Trans Plasma Sci.

[17] Suriyasak C, et al. Alterations of DNA methylation caused by cold plasma treatment restore delayed germination of heat-stressed rice (Oryza sativa L.) seeds. Volume 1. ACS Agricult Sci Technol. 2021;5–10. 1.

[18] Degutytė-Fomins L (2020). Relationship between cold plasma treatment-induced changes in radish seed germination and phytohormone balance. Jpn J Appl Phys.

[19] Liu DX (2010). Main species and physicochemical processes in cold atmospheric-pressure he + O2 Plasmas. Plasma Processes Polym.

[20] Shi H (2017). Reduction of aflatoxin in corn by high voltage atmospheric cold plasma. Food Bioprocess Technol.

[21] Chen X (2022). Degradation efficiency and products of deoxynivalenol treated by cold plasma and its application in wheat. Food Control.

[22] Tolouie H (2021). Argon and nitrogen cold plasma effects on wheat germ lipolytic enzymes: comparison to thermal treatment. Food Chem.

[23] Niemira BA (2012). Cold plasma decontamination of foods. Annual Rev food Sci Technol.

[24] Mir S (2020). Promising applications of cold plasma for microbial safety, chemical decontamination and quality enhancement in fruits. J Appl Microbiol.

[25] Sureshkumar A (2010). Effective bacterial inactivation using low temperature radio frequency plasma. Int J Pharm.

[26] Bourke P (2018). The potential of cold plasma for safe and sustainable food production. Trends Biotechnol.

[27] Ominakhon G (2023). Morphological characteristics and diseases of Soy plants. Eurasian Res Bull.

[28] Cattelan AJ, Dall’Agnol A (2018). The rapid soybean growth in Brazil. OCL.

[29] Anggraini E, Irsan C, GUNAWAN B. Phytophagous insects and predatory arthropods in soybean and zinnia. Biodiversitas J Biol Divers. 2021;22(3):1405–14.

[30] Yildirim A (2022). A breeding study to develop early maturing soybean crosses suitable for double cropping. Turkish J Field Crops.

[31] Kudrin AV, Ivoninsky AV, Eskin VA. Effects of Radiative and Collisional Energy Loss on the Scattering Properties of a Magnetized Plasma Cylinder at the Plasmon Resonances. in 2018 2nd URSI Atlantic Radio Science Meeting *(AT-RASC)*. 2018. IEEE.

[32] Poletti G (2003). Cold plasma treatment of PET fabrics: AFM surface morphology characterisation. Appl Surf Sci.

[33] Boivin R, Kline J, Scime E (2001). Electron temperature measurement by a Helium line intensity ratio method in helicon plasmas. Phys Plasmas.

[34] Lee W (2016). Optical diagnostics with radiation trapping effect in low density and low temperature Helium plasma. Phys Plasmas.

[35] Andrews HB, Sadergaski LR, Myhre KG (2022). Neptunium transition probabilities estimated through laser induced breakdown spectroscopy (LIBS) measurements. J Anal at Spectrom.

[36] Akatsuka H (2019). Optical Emission Spectroscopic (OES) analysis for diagnostics of electron density and temperature in non-equilibrium argon plasma based on collisional-radiative model. Adv Physics: X.

[37] Ferus M (2018). Calibration-free quantitative elemental analysis of meteor plasma using reference laser-induced breakdown spectroscopy of meteorite samples. Astronomy Astrophysics.

[38] Mittal KL. Progress in adhesion and adhesives. Wiley; 2015.

[39] Yu, R., et al., NIST Atomic Spectra Databasehttp://physics.nist.gov/PhysRefData.ASD/index.html, 2006

[40] Ranal MA, Santana DGd (2006). How and why to measure the germination process?. Brazilian J Bot.

[41] Sadeghi H (2011). Effect of seed osmopriming on seed germination behavior and vigor of soybean (Glycine max L). ARPN J Agricultural Biol Sci.

[42] Matthews S (2009). Vigour tests for cabbage seeds using electrical conductivity and controlled deterioration to estimate relative emergence in transplant modules. Seed Sci Technol.

[43] Mancini V, Murolo S, Romanazzi G (2016). Diagnostic methods for detecting fungal pathogens on vegetable seeds. Plant Pathol.

[44] Deng J (2020). Effects of air relative humidity on spectral characteristics of dielectric barrier discharge plasma assisted combustion reactor. Vacuum.

[45] Naeem M (2016). Influence of pulsed power supply parameters on active screen plasma nitriding. Surf Coat Technol.

[46] Goekce S (2016). OES characterization of streamers in a nanosecond pulsed SDBD using N2 and ar transitions. Plasma Sources Sci Technol.

[47] Olenici-Craciunescu S (2009). Characterization of a capillary dielectric barrier plasma jet for use as a soft ionization source by optical emission and ion mobility spectrometry. Spectrochim Acta, Part B.

[48] Bolouki N (2021). Characterizations of a plasma-water system generated by repetitive microsecond pulsed discharge with air, nitrogen, oxygen, and argon gases species. Appl Sci.

[49] Vesel A (2006). Cleaning of porous aluminium titanate by oxygen plasma. Plasma Chem Plasma Process.

[50] Vassallo E (2010). Characterization by optical emission spectroscopy of an oxygen plasma used for improving PET wettability. Vacuum.

[51] Kim B (2002). Improvement of wettability and reduction of aging effect by plasma treatment of low-density polyethylene with argon and oxygen mixtures. J Adhes Sci Technol.

[52] Galmed A, Harith M (2008). Temporal follow up of the LTE conditions in aluminum laser induced plasma at different laser energies. Appl Phys B.

[53] Kramida A, Ralchenko Y, Reader J. NIST atomic spectra database (ver. 5.3). 2015.

[54] Kramida A, Ralchenko Y, Reader J. Team 2015 NIST atomic spectra database (ver. 5.3) National Institute of Standards and Technology, Gaithersburg, MD (Available at: http://physics.nist.gov/asd), 2018.

[55] Mravlje J, Regvar M, Vogel-Mikuš K (2021). Development of cold plasma technologies for surface decontamination of seed fungal pathogens: Present status and perspectives. J Fungi.

[56] Rout S, Srivastav PP. Effect of cold plasma on the technological and functional modification of plant proteins and enzymes. Innovative Food Sci Emerg Technol; 2023:103447.

[57] Wang X (2020). The degradation of Alternaria mycotoxins by dielectric barrier discharge cold plasma. Food Control.

[58] Nasruddin (2015). A simple technique to improve contractile effect of cold plasma jet on acute mouse wound by dropping water. Plasma Processes Polym.

[59] Ekezie F-GC, Sun D-W, Cheng J-H (2019). Altering the IgE binding capacity of king prawn (Litopenaeus Vannamei) tropomyosin through conformational changes induced by cold argon-plasma jet. Food Chem.

[60] Nyaisaba BM (2019). Effects of cold atmospheric plasma on squid proteases and gel properties of protein concentrate from squid (Argentinus ilex) mantle. Food Chem.

[61] Švubová R (2021). Evaluation of the impact of cold atmospheric pressure plasma on soybean seed germination. Plants.

[62] Wahid A (2008). Priming-induced metabolic changes in sunflower (Helianthus annuus) achenes improve germination and seedling growth. Bot Stud.

[63] Lal M, Roy R. Effect of nursery seeding density and fertilizer on seedling growth 1996.

[64] Gao Y (2023). Physiological characteristics of Root Regeneration in Rice Seedlings. Agronomy.

[65] Labelle ER, Kammermeier M (2019). Above-and belowground growth response of Picea abies seedlings exposed to varying levels of soil relative bulk density. Eur J for Res.

[66] Parera V, Parera CA, Feresin GE (2023). Germination and early seedling growth of high Andean native plants under heavy metal stress. Diversity.

[67] Bogale M (2009). Diverse fusarium solani isolates colonise agricultural environments in Ethiopia. Eur J Plant Pathol.

[68] Abd-El-Khair H, El-Gamal G, Nadia. Effects of aqueous extracts of some plant species against Fusarium solani and Rhizoctonia solani in Phaseolus vulgaris plants. Arch Phytopathol Plant Protect. 2011;44(1):1–16.

[69] Horn BW, Dorner JW (2009). Effect of nontoxigenic aspergillus flavus and A. parasiticus on aflatoxin contamination of wounded peanut seeds inoculated with agricultural soil containing natural fungal populations. Biocontrol Sci Technol.

[70] Sivachandiran L, Khacef A (2017). Enhanced seed germination and plant growth by atmospheric pressure cold air plasma: combined effect of seed and water treatment. RSC Adv.

[71] Stolárik T (2015). Effect of low-temperature plasma on the structure of seeds, growth and metabolism of endogenous phytohormones in pea (Pisum sativum L). Plasma Chem Plasma Process.

[72] Shashikanthalu SP, Ramireddy L, Radhakrishnan M (2020). Stimulation of the germination and seedling growth of cuminum cyminum L. seeds by cold plasma. J Appl Res Med Aromatic Plants.

[73] Los A (2019). Investigation of mechanisms involved in germination enhancement of wheat (Triticum aestivum) by cold plasma: effects on seed surface chemistry and characteristics. Plasma Processes Polym.

[74] Mravlje J (2021). Cold plasma affects germination and fungal community structure of buckwheat seeds. Plants.

[75] Bol’Shakov A (2004). Radio-frequency oxygen plasma as a sterilization source. AIAA J.

[76] Lu B-J (2021). The impact of air or nitrogen non-thermal plasma on variations of natural bioactive compounds in Djulis (Chenopodium Formosanum Koidz.) Seed and the potential effects for human health. Atmosphere.

[77] Thirumdas R (2018). Plasma activated water (PAW): Chemistry, physico-chemical properties, applications in food and agriculture. Trends Food Sci Technol.

[78] Mai-Prochnow A (2014). Atmospheric pressure plasmas: infection control and bacterial responses. Int J Antimicrob Agents.

[79] Tamošiūnė I (2020). Cold plasma treatment of Arabidopsis thaliana (L.) seeds modulates plant-associated microbiome composition. Appl Phys Express.

[80] Berardinelli A (2021). Features and application of coupled cold plasma and photocatalysis processes for decontamination of water. Chemosphere.

[81] Ohta T. Plasma in agriculture Cold plasma in food and agriculture, 2016:205–221.

[82] Dogan C (2019). Sterilization of natural rose water with nonthermal atmospheric pressure plasma. Arab J Sci Eng.

[83] Gao X (2019). Effect of dielectric barrier discharge cold plasma on pea seed growth. J Agric Food Chem.

[84] Fridman A (2008). Plasma chemistry.

[85] Talbi S (2015). Drought tolerance in a Saharian plant Oudneya Africana: role of antioxidant defences. Environ Exp Bot.

[86] Ji S-H (2016). Effects of high voltage nanosecond pulsed plasma and micro DBD plasma on seed germination, growth development and physiological activities in spinach. Arch Biochem Biophys.

[87] Zhou R (2016). Effects of atmospheric-pressure N2, he, air, and O2 microplasmas on mung bean seed germination and seedling growth. Sci Rep.

[88] Pańka D (2022). Can cold plasma be used for boosting plant growth and plant protection in sustainable plant production?. Agronomy.

[89] Fadhlalmawla SA (2019). The impact of cold atmospheric pressure plasma jet on seed germination and seedlings growth of fenugreek (Trigonella foenum-graecum). Plasma Sci Technol.

[90] Ling L (2015). Cold plasma treatment enhances oilseed rape seed germination under drought stress. Sci Rep.

[91] Meiqiang Y (2005). Stimulating effects of seed treatment by magnetized plasma on tomato growth and yield. Plasma Sci Technol.

[92] Sookwong P (2014). Application of oxygen-argon plasma as a potential approach of improving the nutrition value of pre-germinated brown rice. J Food Nutr Res.

[93] Almarashi JQ (2023). Second grounded electrode non-equilibrium atmospheric pressure argon plasma jet impact on germination of basil (Ocimum basilicum) seeds. J Taibah Univ Sci.

[94] Lotfy K, Al-Harbi NA, Abd El-Raheem H (2019). Cold atmospheric pressure nitrogen plasma jet for enhancement germination of wheat seeds. Plasma Chem Plasma Process.

[95] Guragain RP et al. Germination enhancement of mustard (Brassica nigra) seeds using dielectric barrier discharge (DBD). AIP Adv, 2023:13(3):1–13.

[96] Guragain RP et al. Non-Thermal Plasma: A Promising Technology for the Germination Enhancement of Radish (Raphanus sativus) and Carrot (Daucus carota sativus L.) J Food Qual. 2023;2023:1–15.

[97] Sheteiwy MS (2019). Cold plasma treatment and exogenous salicylic acid priming enhances salinity tolerance of Oryza sativa seedlings. Protoplasma.

[98] Taheri S, Brodie G, Gupta D (2019). Heat uniformity study and viability of red lentil at different seed moisture contents after low-dose microwave treatment. Trans ASABE.

[99] Mitra A (2014). Inactivation of surface-borne microorganisms and increased germination of seed specimen by cold atmospheric plasma. Food Bioprocess Technol.

[100] Velichko I (2019). Plasma jet and dielectric barrier discharge treatment of wheat seeds. Plasma Chem Plasma Process.

[101] Guragain RP (2021). Impact of plasma-activated water (PAW) on seed germination of soybean. J Chem.

[102] Guragain RP (2023). Improvements in germination and growth of sprouts irrigated using plasma activated water (PAW). Water.

[103] Silva A, et al. Effect of Atmospheric pressure Cold plasma gliding Arc Discharge Remote Treatment on Microbiological and Physicochemical Properties of Black Peppercorns (Piper nigrum). 2022.

[104] Meng Y (2017). Enhancement of germination and seedling growth of wheat seed using dielectric barrier discharge plasma with various gas sources. Plasma Chem Plasma Process.

[105] Guo Q (2018). Improvement of wheat seed vitality by dielectric barrier discharge plasma treatment. Bioelectromagnetics.

[106] Li Y (2017). Air atmospheric dielectric barrier discharge plasma induced germination and growth enhancement of wheat seed. Plasma Chem Plasma Process.

[107] Hou F, Thseng F (1991). Studies on the flooding tolerance of soybean seed: varietal differences. Euphytica.

[108] Bouslama M, Schapaugh W (1984). Stress tolerance in soybeans. I. evaluation of three screening techniques for heat and drought tolerance 1. Crop Sci.

[109] Singh B (2017). Developing a screening tool for osmotic stress tolerance classification of rice cultivars based on in vitro seed germination. Crop Sci.

[110] Hussain S (2016). Physiological and biochemical mechanisms of seed priming-induced chilling tolerance in rice cultivars. Front Plant Sci.

[111] Yigit N (2016). Determination of the effect of drought stress on the seed germination in some plant species. Water Stress in Plants.

[112] Ivankov A (2021). The effects of red clover seed treatment with cold plasma and electromagnetic field on germination and seedling growth are dependent on seed color. Appl Sci.

[113] Volin JC (2000). Modification of seed germination performance through cold plasma chemistry technology. Crop Sci.

[114] Yodpitak S (2019). Cold plasma treatment to improve germination and enhance the bioactive phytochemical content of germinated brown rice. Food Chem.

[115] Starič P, Grobelnik Mlakar S, Junkar I (2021). Response of two different wheat varieties to glow and afterglow oxygen plasma. Plants.

[116] Zhang B, Li R, Yan J (2018). Study on activation and improvement of crop seeds by the application of plasma treating seeds equipment. Arch Biochem Biophys.

[117] Su L (2016). Reactive oxygen species induced by cold stratification promote germination of Hedysarum scoparium seeds. Plant Physiol Biochem.

[118] Shelar A (2022). Emerging cold plasma treatment and machine learning prospects for seed priming: a step towards sustainable food production. RSC Adv.

[119] Li K (2021). Cold plasma seed treatment improves chilling resistance of tomato plants through hydrogen peroxide and abscisic acid signaling pathway. Free Radic Biol Med.

[120] Mildaziene V (2022). Biochemical and physiological plant processes affected by seed treatment with non-thermal plasma. Plants.

[121] Ghodsimaab SP (2023). Scanning electron microscopy, biochemical and enzymatic studies to evaluate hydro-priming and cold plasma treatment effects on the germination of Salvia Leriifolia Benth. Seeds. Front Plant Sci.

[122] Adhikari B (2020). Cold plasma seed priming modulates growth, redox homeostasis and stress response by inducing reactive species in tomato (Solanum lycopersicum). Free Radic Biol Med.

[123] Xu Z (2022). Study on immediate and long-term growth inhibition of Microcystis aeruginosa by non-thermal plasma. Chem Eng J.

[124] Ahmed N (2023). A study to examine the ageing behaviour of cold plasma-treated agricultural seeds. Sci Rep.

[125] de Groot GJ (2018). Cold plasma treatment for cotton seed germination improvement. Sci Rep.

[126] Mutete P (2019). Hyphaene petersiana dormancy and germination. Seed Sci Technol.

[127] Daeschlein G (2010). In vitro killing of clinical fungal strains by low-temperature atmospheric-pressure plasma jet. IEEE Trans Plasma Sci.

[128] Filatova I, et al. Fungicidal effects of plasma and radio-wave pre-treatments on seeds of grain crops and legumes. Plasma Bio Decontamination Med Food Secur. 2011:469–79.

[129] Ouf SA, Mohamed AAH, El-Sayed WS (2016). Fungal decontamination of fleshy fruit water washes by double atmospheric pressure cold plasma. CLEAN–Soil Air Water.

[130] Sun P et al. Atmospheric pressure cold plasma as an antifungal therapy. Appl Phys Lett, 2011;98(2):501–3.

[131] Punia Bangar S (2022). Cold plasma for microbial safety: Principle, mechanism, and factors responsible. J Food Process Preserv.

[132] Lee Y (2021). Enhancement of seed germination and microbial disinfection on ginseng by cold plasma treatment. J Ginseng Res.

[133] Gupta A, Nanda V, Singh B. Cold plasma for food processing. Food Sci Technol, 2017:623–60.

[134] Nehra V, Kumar A, Dwivedi H (2008). Atmospheric non-thermal plasma sources. Int J Eng.

[135] Jakob H, Kim MK (2020). Generation of non-thermal plasmas over large and complex surfaces. Plasma Res Express.

[136] Boekema B (2015). A new flexible DBD device for treating infected wounds: in vitro and ex vivo evaluation and comparison with a RF argon plasma jet. J Phys D.

[137] Schneider J (2005). Investigation of the practicability of low-pressure microwave plasmas in the sterilisation of food packaging materials at industrial level. Surf Coat Technol.

[138] Dong J (2020). Contactless flash sintering based on cold plasma. Scripta Mater.

[139] Yepez X (2022). Recent advances and potential applications of atmospheric pressure cold plasma technology for sustainable food processing. Foods.

[140] Özdemir E, et al. Cold plasma application to fresh green leafy vegetables: impact on microbiology and product quality. Comprehens Rev Food Sci Food Safe. 2023: 1–32. 10.1111/1541-4337.13231 37661766

[141] Ruzic R, Jerman I, Gogala N (1998). Effects of weak low-frequency magnetic fields on spruce seed germination under acid conditions. Can J for Res.

[142] Nayab N et al. Cold Plasma Based Enhancement of Colonization of Plant Growth Promoting Bacteria (PGPB) and Fungal Resistance of Seeds 2022.

[143] Ansari A, Parmar K, Shah M (2022). A comprehensive study on decontamination of food-borne microorganisms by cold plasma. Food Chemi Mol Sci.

